# Implementing Sustainable Mobile Health Technology to Optimize a Smoking Cessation Program for Lao People With HIV (Project I-STOP): Protocol for a Hybrid Type-2 Pragmatic Effectiveness-Implementation Study

**DOI:** 10.2196/100518

**Published:** 2026-10-05

**Authors:** Phonepadith Xangsayarath, Cate Moriasi, Phayvanh Keopaseuth, Khatthanaphone Phandouangsy, Khanti Thongkham, Bouathong Simanovong, Michael S Businelle, Steven K Sutton, Margaret M Byrne, Kea L Turner, Jennifer I Vidrine, Khue-Tu T Doan, Chanthavy Soulaphy, Kanchana Thilakoun, Sarah R Jones, Thanh Cong Bui, Damon J Vidrine

**Affiliations:** 1 Department of Communicable Disease Control, Ministry of Health of Lao People's Democratic Republic, Vientiane Capital, Lao People's Democratic Republic; 2 Ministry of Health of Lao People's Democratic Republic, Vientiane Capital, Lao People's Democratic Republic; 3 Secretariat of the National Tobacco Control Committee, Ministry of Health of Lao People's Democratic Republic, Vientiane Capital, Lao People's Democratic Republic; 4 National Center for HIV/AIDS and STIs (CHAS), Ministry of Health of Lao People's Democratic Republic, Vientiane Capital, Lao People's Democratic Republic; 5 Department of Family and Preventive Medicine, College of Medicine, University of Oklahoma Health Campus, Oklahoma City, OK, United States; 6 Department of Biostatistics and Bioinformatics, Moffitt Cancer Center, Tampa, FL, United States; 7 Department of Health Outcomes and Behavior, Moffitt Cancer Center, Tampa, FL, United States; 8 Division of Health Systems, Policy, and Leadership Innovations, University of North Carolina at Chapel Hill School of Nursing, Chapel Hill, NC, United States; 9 Lineberger Comprehensive Cancer Center, University of North Carolina at Chapel Hill, Chapel Hill, NC, United States; 10 TSET Health Promotion Research Center, Stephenson Cancer Center, University of Oklahoma Health Campus, 655 Research Parkway, Suite 400, Oklahoma City, OK, 73104, United States, 1 7134746040

**Keywords:** tobacco treatment, implementation science, mobile health, people with HIV, Lao People's Democratic Republic, mHealth, mobile phone

## Abstract

**Background:**

Tobacco use remains the leading modifiable risk factor for causing cancer worldwide, particularly among people with HIV. In Laos, 61%‐80% of male people with HIV and 3%‐10% of female people with HIV smoke cigarettes. They currently have no theoretically and empirically based smoking cessation support. Our team developed a scalable and affordable mHealth (mobile health)–based automated treatment program to support Lao and Cambodian smokers to quit smoking. We also pioneered the Ask-Advise-Connect approach to identify patients who smoke and to connect them to treatment.

**Objective:**

This hybrid type-2 pragmatic effectiveness-implementation study aims to compare 2 smoking cessation implementation strategies in 8 antiretroviral therapy (ART) clinics in the 6 most populous regions across Laos, using a parallel cluster randomized trial design.

**Methods:**

We will compare an Ask-Advice-Connect approach paired with an mHealth-based automated treatment program (AA-MAP) with an Ask-Advice-Connect approach paired with less resource-intensive printed self-help material (AA-SH). To guide assessment of implementation determinants and outcomes, we use the Practical, Robust Implementation and Sustainability Model framework. Aim 1 is to evaluate the reach and effectiveness of AA-MAP vs AA-SH. Reach is the proportion of people with HIV who smoke and are willing to make a quit attempt that enroll in treatment. Effectiveness is the proportion of enrolled participants (n=up to 1200) who achieve biochemically confirmed point prevalence abstinence 6 months after enrollment. We hypothesize that compared with AA-SH, AA-MAP will have a lower reach but will be more effective. We will also estimate the real-world impact (impact = reach × effectiveness) of each intervention. Aim 2 is to evaluate other implementation outcomes (eg, adoption, implementation fidelity, and sustainability) and identify implementation determinants of AA-MAP and AA-SH in the ART clinic setting using mixed methods. Aim 3 is to conduct a comprehensive assessment of the resource use and costs of implementing AA-MAP and AA-SH and calculate the absolute and relative cost-effectiveness of the 2 intervention strategies.

**Results:**

The study has been funded since August 2024. This study was approved by the ethical review boards of the Lao Ministry of Health–National Ethics Committee for Health Research and the University of Oklahoma Health Campus. The Multiple Principal Investigators met with the selected ART clinics. As of September 2026, we have launched the implementation of AA-SH and AA-MAP at 4 ART clinics. We plan to expand the implementation at the other ART clinics by December 2026.

**Conclusions:**

This project will contribute important actionable inputs that will inform the influential Lao National Tobacco Control Committee and Ministry of Health to implement the strategies in diverse hospital settings in future large-scale hybrid type II/III trials. Ultimately, our course of research can transform health care delivery and contribute to reducing tobacco-related morbidities and mortalities in Laos.

## Introduction

Tobacco use remains the leading modifiable risk factor for causing cancer worldwide [1]. Of approximately 7.4 million citizens in Laos [2], 44%‐51% of men and 7% of women smoke tobacco [3,4], making the smoking prevalence in Laos the second highest among Southeast Asian countries [5]. Following the World Health Organization’s (WHO) MPOWER framework (monitor tobacco use and prevention policies; protect people from tobacco smoke; offer help to quit tobacco use; warn about the dangers of tobacco; enforce bans on tobacco advertising, promotion, and sponsorship; and raise taxes on tobacco) [6], the National Tobacco Control Committee (NTCC) of Laos has implemented several tobacco control measures (eg, taxing tobacco products, expanding smoke-free environments, and mandating package warnings and comprehensive bans on tobacco advertising). However, neither a national tobacco treatment program nor smoking cessation support in hospitals or clinics is available [3,7]. In addition, funding limitations restricted a past effort by the NTCC to train and retain enough counselors to scale up a quitline with telephone counseling that was piloted at the national Mahosot Hospital. Thus, establishing and implementing scalable evidence-based smoking cessation interventions is a priority of the Lao NTCC and Lao Ministry of Health (MOH) for cancer and tobacco-induced disease control.

Tobacco use is particularly harmful for people with HIV [8]. A compelling body of evidence from high-income countries indicates that tobacco smoking is among the leading causes of morbidity and mortality in people with HIV [8-15]. For example, lung cancer rates are significantly higher in smokers who are HIV-positive compared with smokers who are HIV-negative [16-20]. People with HIV who smoke, compared with people with HIV who do not smoke, also have higher risks of renal diseases [12], cardiovascular diseases, decreased bone mineral density and fracture, chronic obstructive pulmonary disease [9,11], opportunistic and nonopportunistic infections, periodontal diseases, and poor quality of life (eg, physical or cognitive functioning) [10,12]. Smoking also negatively affects antiretroviral therapy (ART), leading to poor viral and immunologic response [21,22]. The mortality rate for people with HIV who smoke is twice the mortality rate of people with HIV who do not smoke [14,23], and the estimated population attributable risk of all-cause mortality associated with smoking in people with HIV ranges from 24%‐62% [13,24].

Worldwide, the prevalence of smoking among people with HIV is 1.6‐3 times higher than that in the general population [8,25-30]. Data from the National Center for HIV/AIDS and STIs of Laos (CHAS) indicate that 61%‐80% of male people with HIV and 3%‐10% of female people with HIV smoke cigarettes [31]. Despite the high prevalence of smoking among people with HIV, there is no effort to monitor tobacco use among people with HIV and no available program to treat tobacco use. Consequently, people with HIV in Laos currently have no theoretically and empirically based smoking cessation support. Thus, implementing sustainable and evidence-based smoking cessation interventions for people with HIV in Laos is critically needed.

Funded by the US National Cancer Institute (NCI) and Fogarty International Center, our studies are the first to use scalable and affordable mHealth (mobile health) technology to provide fully automated cessation treatment for the general patient population (R33CA253600) and for survivors and caregivers of cancer (R21CA253600-02S1) in Laos. Our mHealth-based automated treatment program (MAP) involves interactive, tailored, personalized content (text messages with photos and videos) delivered via a smartphone app. In addition, we are currently collaborating with the Cambodian MOH to evaluate a similar mHealth intervention tailored to people with HIV who smoke (U01CA261598) [32]. The results, to date, indicate that participant engagement and retention are excellent and the digital platform supporting our app shows robust functionality [33,34]. In addition to our work, there is substantial evidence that mobile phone–based smoking cessation interventions are effective [35,36], cost-effective [1,37], and affordable for use in the lowest-income countries [38-40]. Thus, app-based cessation treatment represents an ideal way to treat people with HIV who smoke in Laos. Laos is among the lowest-income countries [41], but 100% of the Lao urban districts and 96% of the rural villages are covered by a mobile-cellular network [42]. These statistics suggest that Laos has an ideal infrastructure for implementing an mHealth-based smoking cessation treatment program on a large scale.

Along with effective cessation treatments, it is imperative to implement procedures to improve the identification of patients who smoke and to facilitate connections to treatment. One such approach pioneered by our team is Ask-Advise-Connect (asking patients about smoking at every visit, briefly advising those who smoke to quit, and connecting them to treatment), which has demonstrated great impact in our US studies [26,43,44].

This protocol paper describes the design and methods to compare 2 smoking cessation implementation strategies for Lao people with HIV in 8 ART clinics in the 6 most populous provinces or regions across Laos. To gain insight into both the effectiveness of our MAP and critical implementation outcomes, we use a hybrid type-2 pragmatic effectiveness-implementation study and a parallel cluster randomized trial design. Specifically, we compare an Ask-Advice-Connect approach paired with our previously developed MAP (AA-MAP) to an Ask-Advice-Connect approach paired with less resource-intensive printed self-help material (AA-SH) to determine which implementation strategy performs better. To guide our implementation work, we use the Practical, Robust Implementation and Sustainability Model (PRISM) [45-47], which expands the RE-AIM (Reach, Effectiveness, Adoption, Implementation, and Maintenance) framework to include more key, multilevel contextual factors relevant to program implementation throughout all stages from planning through sustainment.

This project capitalizes on strong and equitable partnerships among researchers at the University of Oklahoma Health Campus, Moffitt Cancer Center, University of North Carolina at Chapel Hill, Lao NTCC, and Lao MOH. The team consists of experts in tobacco treatment, mHealth, implementation science, HIV, economic evaluation, and health care administration. The project has the potential to transform HIV care delivery throughout the country and to reduce tobacco-related cancers and other morbidities in Laos.

## Methods

### Project Overview

This hybrid type-2 pragmatic effectiveness-implementation study aims to evaluate the AA-SH and AA-MAP implementation strategies for smoking cessation at 8 ART clinics in the 6 most populous provinces or regions across Laos, using a parallel cluster randomized controlled trial (RCT) design. Aim 1 is to evaluate the reach and effectiveness of the AA-SH and AA-MAP approaches. We hypothesize that the less resource-intensive AA-SH will have greater reach than AA-MAP (90% vs 70%, respectively, based on our previous and ongoing studies [26,32,34]); and that the interactive tailored AA-MAP will have greater effectiveness than AA-SH (16% vs 8%, respectively). We will also estimate the real-world impact (impact = reach × effectiveness) of each intervention. Aim 2 is to use mixed methods to evaluate additional implementation outcomes at the patient level (eg, adherence to AA-MAP), ART clinic level (eg, implementation fidelity and sustainability), and national levels (ie, external factors such as MOH policies, resources, and incentives). Aim 3 is to conduct a comprehensive assessment of the resource use, implementation costs, and the absolute and relative cost-effectiveness of the 2 strategies.

### Ethical Considerations

The Lao National Ethics Committee for Health Research reviewed and approved all of the research materials (including study protocol, informed consent form, questionnaires, and intervention content) for all participating sites in Laos (68). The Institutional Review Board (IRB) of the University of Oklahoma Health Campus serves as the single IRB of record and approved all of the study for all participating US domestic institutions (17386). The currently approved IRB protocol version is dated December 22, 2025. All selected ART clinics will undergo an informed consent process, which will be conducted in Lao language by the Lao Senior Research Coordinators. One of the Multiple Principal Investigators or Lao coinvestigators will also be present during the informed consent process to answer any questions that the clinic leaders may have. During the process, the clinic directors or leaders will be informed of the nature of the investigation, the steps that the clinics will complete, and the types of interventions involved. The IRB-approved bilingual consent form also explains how information related to study participation will be handled, including data management and plans to publish data in group format without identifying information, either at the patient or clinic levels. The clinic leaders will be made aware that the clinic should provide the resources needed to implement the assigned smoking cessation strategy (eg, staff time to perform AA-SH or AA-MAP, which may take 1‐5 minutes for each ART patient). The clinic leaders will provide written informed consent for the respective clinic to participate. When invited to participate, patient participants, staff participants, and key informants at each selected clinic will also undergo a separate, individual informed consent process, conducted in Lao language by the local research staff. All individuals who choose to participate in this study will provide written informed consent. For data collection for research purposes, the clinics and clinic staff will be compensated for the time and other resources dedicated to research data collection.

### Main Study Design

#### Overview

We use a hybrid type-2 pragmatic effectiveness-implementation study design and a parallel cluster RCT design (Figure 1) to evaluate the AA-SH and AA-MAP implementation strategies for smoking cessation at 8 ART clinics in the 6 most populous provinces or regions across Laos. We used the CONSORT (Consolidated Standards of Reporting Trials) [48] and SPIRIT (Standard Protocol Items: Recommendations for Interventional Trials) [49] checklists in the preparation of this paper.

**Figure 1.**
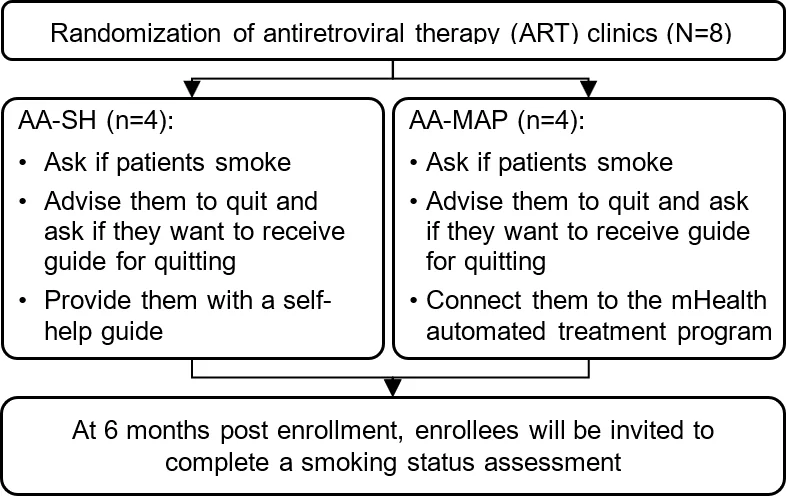
Trial schema. AA-MAP: Ask-Advice-Connect–mHealth-based automated treatment program; AA-SH: Ask-Advice-Connect–self-help material; ART: antiretroviral therapy; mHealth: mobile health.

#### Design Considerations

Several aspects of this project were carefully considered before deciding on the proposed design. We chose a cluster (vs individual) RCT design as it is a robust, pragmatic design for service interventions (AA-SH and AA-MAP) at the cluster (ART clinics) level rather than the patient level. We considered a stepped-wedge design, which would have allowed all clinics to receive both interventions during this trial and within-cluster comparisons. However, the drawbacks of this design are the risk of unequal chronological exposure to interventions, and the potential risk of internal contamination due to carry-over effects (ie, during the treatment period, enrollees may be exposed to the other intervention if the clinic switched interventions). Thus, we chose the parallel cluster RCT design but will allow all clinics the option to implement both AA-SH and AA-MAP after this trial’s period.

#### Conceptual Frameworks

To guide our implementation work, we have been using and will continue to use the PRISM [45-47]. We selected PRISM because it includes the RE-AIM framework, which helps to measure the desired outcomes of AA-SH and AA-MAP (ie, reach and effectiveness) in our previous studies and in aim 1 of this study. PRISM expands RE-AIM to include more key, multilevel contextual factors relevant to program implementation and sustainment, particularly in adopting technology-based interventions in clinical settings.

We use the phase-based model (PBM), a theoretical framework that is specific to smoking cessation [50], to guide our MAP treatment. PBM partitions the cessation process into 4 phases: motivation, preparation (precessation), cessation (quit date to 2 weeks post quit), and maintenance (up to 6 months post quit). PBM helps to identify challenges or opportunities at each phase, explains underlying phase-specific mechanisms, and facilitates selection of intervention components and measures. Among several putative mechanisms, some are relevant across phases and have been empirically shown to be reliably associated with long-term abstinence. These include withdrawal and craving, motivation to quit, positive or negative affect, coping with stress and urges, self-efficacy, and perceived support [50-54]. As such, MAP specifically targets these mechanisms.

#### Study Sites and Setting

It is estimated that Laos had 17,000 people with HIV in 2022 (female=38%) [55]. The number of new cases were 1550 in 2021 and 1748 in 2022 [31]. Of those diagnosed, 97% of them are registered with an ART clinic, and 77% of them are on ART. Of those on ART, 96% of patients are aged ≥18 years, and the mean duration on ART is 6.6 years [31]. Registered patients have periodic prescheduled appointments (about once per month for new patients and once per 4 months for patients with controlled viral load).

Of the 11 main ART clinics in Laos, CHAS recommends implementing this project at 8 ART clinics in the 6 provinces or regions that are most populous or have an increasing HIV+ incidence. Besides currently registered patients, these clinics together gain approximately 1500 new patients each year [31]. These clinics provide comprehensive care for registered ART patients, including ART, ART adherence counseling, nutrition, risk-reduction counseling, and treatments for comorbidities other than tobacco use. To avoid potential skewness, we created 2 sets of 4 clinics with comparable patient populations and available resources and then randomly assigned each set to an implementation strategy (AA-SH or AA-MAP).

#### Training ART Clinic Staff

All potential implementers will receive an initial 2-hour in-person group training on implementing Ask-Advice-Connect and collecting data using REDCap. The training also includes information on why people with HIV should quit smoking, the effectiveness of the Ask-Advice-Connect approach in high-income countries, health care provider roles, available nicotine replacement therapies (NRTs) in Laos, and time for questions and answers. The initial training will be held immediately before the launch of the AA-SH or AA-MAP implementation at the ART clinics and will emphasize the importance of fidelity to the interventions. The initial training will be video recorded for future review and for the training of newly hired clinic staff. There will be 4 “booster” training sessions at months 1, 6, 12, and 18 to reinforce the information in the initial training, address emerging issues, and answer clinic staff’s questions. These 30-minute booster sessions will coincide with required clinic-wide staff meetings. Both the initial and booster training sessions will involve didactic lectures and role-playing exercises.

#### Implementing the Interventions (See Recruitment Flow)

##### AA-SH Strategy

In clinics assigned to AA-SH, staff will ask the patient at every visit if they smoke, advise smokers to quit, ask if they want to receive help with quitting, and provide them with a self-help guide Figure 2. The AA-SH will be performed by counselors (counseling sessions occur at almost every visit and counselors have more time with patients) or nurses (when vital signs or blood samples are collected). It is optional for physicians to provide additional brief advice to quit (because physicians have a more authoritative voice in Asian culture but less time with patients). We will document which types of health care professionals provide advice to quit for additional analysis. The clinic staff will use a short form attached to patients’ paper medical record to perform AA-SH, with a standardized question about smoking status (“Did you smoke cigarettes in the past 7 days? [Yes/No]”), a sample script to briefly advise smokers to quit, and 2 standardized questions about intent to quit (“Are you willing to make a quit attempt in the next 2 weeks? [Yes/Remind me later],” and if yes: “Do you want to receive more information regarding how to quit? [Yes/No]”). Those who answer yes to both questions will be provided with the self-help guide. The ART clinic staff will also ask patients if they consent to be contacted at 6 months by research staff. The self-help guide is a 22-page booklet that we developed in our R21/R33 study (Figure 3). Attached to the guide is an information sheet regarding NRT (eg, patches and lozenges), where to buy them, and how to use them.

**Figure 2.**
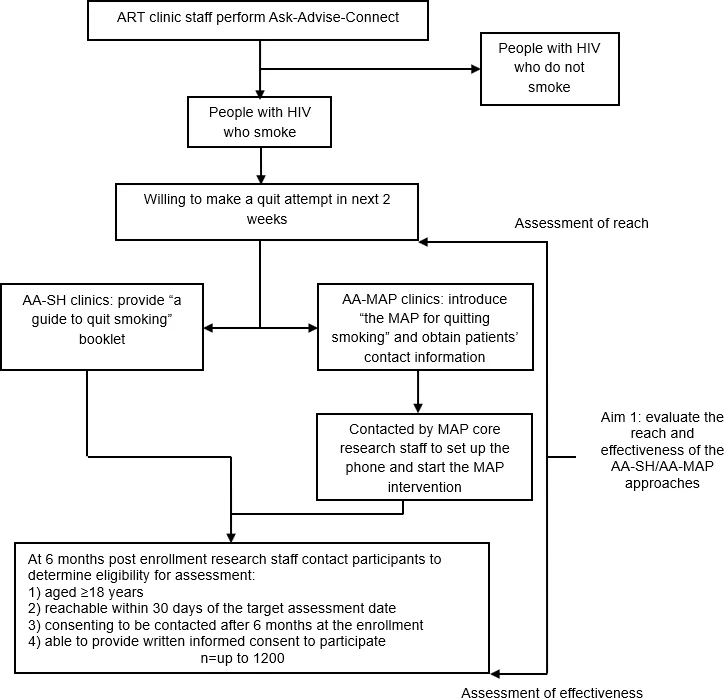
CONSORT (Consolidated Standards of Reporting Trials) flow diagram. AA-MAP: Ask-Advice-Connect–mHealth-based automated treatment program; AA-SH: Ask-Advice-Connect–self-help material; ART: antiretroviral therapy; MAP: mHealth-based automated treatment program.

**Figure 3.**
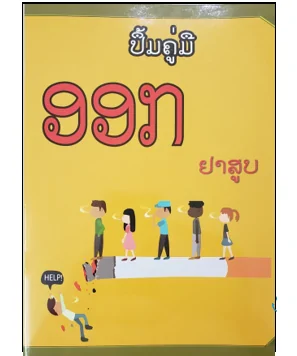
Self-help guide.

##### AA-MAP Strategy

In the AA-MAP arm, clinic staff will do the ask-advise steps, and then will ask if smokers use a smartphone and proactively connect them to the MAP. Specifically, clinic staff will show a QR code (linked to a brief REDCap form) to smokers, and guide them through scanning the QR code, answering the few questions on the landing page (about their names and contact information), and submitting the form. Through our ongoing studies in Laos, NCLE (National Center for Laboratory and Epidemiology) and NTCC staff members have been proficient at managing the mHealth platform and app (ie, with minimal help from US investigators); these will serve on the MAP core team to provide technical assistance (eg, app installation and troubleshooting) to all end users. Within 48 hours of receiving the REDCap form, MAP core staff will communicate with the enrollees (via phone calls and/or text messages) to instruct them to download, sign up, and use the smoking cessation app (named Insight) [56]. An instructional video of these steps will also be sent to the enrollees. For smokers who do not have a smartphone, the clinic staff will explain that the smoking cessation intervention will be available to them in the future.

Our smoking cessation app (Figure 4) is fully automated and interactive. Its content is adapted from the team’s previous efforts and from the WHO- and US NCI–suggested message banks [57]. MAP is designed to tap the theoretical mechanisms described in the PBM (see the Conceptual Frameworks section). That is, treatment content is designed to increase motivation, self-efficacy, use of coping skills and social support, and to reduce nicotine withdrawal symptoms and stress. The mHealth treatment will begin immediately after enrollment and will continue for 26 weeks. MAP allows for several levels of personalization and tailoring for each participant. First, on day 1, participants will be asked some questions about their biological sex, past quit attempts, and the presence or fear of specific comorbidities. Messages tailored to these responses will be delivered automatically throughout the treatment period. Second, there will be different bins of messages for different cessation phases to ensure that the intervention content targets critical mechanisms of each phase. Phase identification follows the PBM’s suggestion: preparation (enrollment to quit date), cessation (quit date to 4 weeks post-quit date), and maintenance (4‐26 weeks post-quit date) [58]. Finally, the app will prompt participants to complete a brief (4‐6 items) assessment every week (eg, stress or self-efficacy levels); answers to these questions will drive the delivery of appropriate content to each participant.

**Figure 4.**
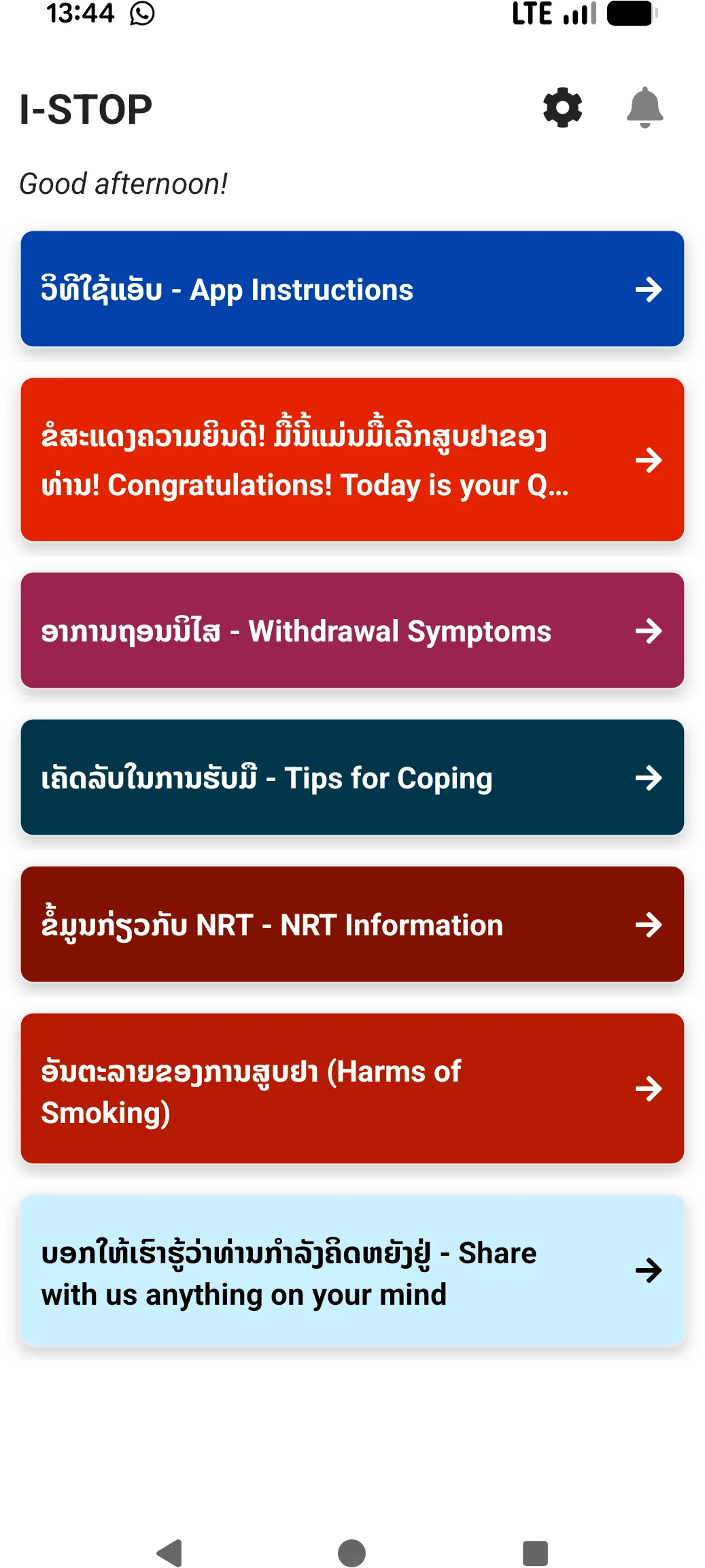
Our smoking cessation app. LTE: Long Term Evolution; NRT: nicotine replacement therapy.

##### Other Strategies to Ensure Treatment and Implementation Fidelity

Besides the standardized trainings, we will use other strategies as recommended by the NIH Behavior Change Consortium [59] and Carroll’s conceptual framework for implementation fidelity [60] to maximize fidelity across multiple clinics. These include all clinics following a standardized protocol, building the standardized Ask-Advice-Connect component into the medical questionnaire, and monitoring AA-SH and AA-MAP enrollment in the REDCap.

### Other Study Components

Besides the main study component (ie, the cluster RCT with patient participants to evaluate the reach and effectiveness of the interventions in aim 1), we will use a mixed methods research approach in aim 2 to assess other PRISM-based implementation outcomes (eg, adoption, implementation fidelity, and sustainability) and to identify key determinants associated with these outcomes (eg, intervention characteristics, patient characteristics, organization characteristics, or external factors) [45,61]. We will use the convergent mixed methods design, in which quantitative and qualitative data are collected separately and then merged in analysis for interpretation [62]. Therefore, in aim 2, there will be 3 other study components: clinic staff survey, in-depth interview (IDI), and key informant interview (KII). In the clinic staff survey component, clinic staff will participate in a self-administered survey intended to assess implementation outcomes of AA-MAP and AA-SH. To further assess implementation outcomes, face-to-face IDIs with ART clinic staff will be conducted to determine staff experiences with implementing the smoking cessation programs. Finally, face-to-face KIIs will be conducted with stakeholders whose decisions affect the functioning of the ART clinics.

### Participants and Eligibility

#### Inclusion Criteria

Inclusion criteria for patient participants (ie, people with HIV who smoke) in the cluster RCT for the assessment at 6 months are the following: (1) being aged ≥18 years; (2) reachable within 30 days of the target assessment date by different methods (up to 4 phone calls on different days and times, 3 text messages for each platform [eg, SMS, Telegram, or WhatsApp], 3 emails if applicable, and via direct contact at a prescheduled clinic appointment if applicable), (3) consenting (at the time of enrollment) to be contacted at 6 months post enrollment, and (4) able to provide a written informed consent at month 6 when contacted by the research staff to participate.

Participants and inclusion criteria for other study components are the following: (1) being aged ≥18 y, (2) employed by the clinic or institution for at least 3 months, and (3) able to provide written informed consent to participate (Table 1).

**Table 1.** Inclusion criteria for different study components.

Study component	Participants	Inclusion criteria
Patient assessment (n=up to 1200)	People with HIV who accepted smoking cessation treatment	Aged ≥18 years Reachable within 30 days of the target assessment date Consenting to be contacted at 6 months post enrollment Able to provide written informed consent to participate
Clinic staff survey (n=up to 200)	ART^a^ clinic staff involved in performing Ask-Advice-Connect	Aged ≥18 years Employed by the clinic/institution for at least three months Able to provide written informed consent to participate
In-depth interview (n=25‐40)	ART clinic staff involved in performing Ask-Advice-Connect	Aged ≥18 years Employed by the clinic/institution for at least three months Able to provide written informed consent to participate
Key informant interview (n=up to 20)	Key informants: ART clinic directors or leaders Key stakeholders of the hospitals (that house ART clinics) or provincial health departments CHAS^b^ technical officers and authorities NTCC^c^ stakeholders Lao MOH^d^ stakeholders	Aged ≥18 years Employed by the clinic/institution for at least three months Able to provide written informed consent to participate

a ART: antiretroviral therapy.

b CHAS: Center for HIV/AIDS and STIs of Laos.

c NTCC: National Tobacco Control Committee.

d MOH: Ministry of Health.

#### Exclusion Criteria

There were no exclusion criteria for this protocol.

### Measures

#### Assessment of Reach

People with HIV who smoke and are willing to make a quit attempt within the next 2 weeks will be offered treatment (self-help guide or MAP). Reach is the proportion of those who enroll in treatment. There are >16,000 registered patients at the 8 ART clinics. Given the male and female ratio and estimated smoking prevalence by sex, we estimate that 5760 male people with HIV and 450 female people with HIV are current smokers. Our previous and ongoing studies in Cambodia show that 65%‐80% of people with HIV who smoke are willing to make a quit attempt and enroll in treatment [34]. Conservatively assuming that only 50% (3105/6210) of Lao people with HIV who smoke agree to be enrolled in AA-SH or AA-MAP, there will be at least 2880 male and at least 225 female participants. Of these, based on our previous studies and the rate of smartphone ownership among Lao people with HIV, we predict that the reach of AA-SH will be 90% and the reach of AA-MAP will be 70%.

#### Brief Weekly Smartphone Assessments

Participants in the AA-MAP arm are asked to answer some questions on day 1 and to complete brief weekly assessments (4‐6 questions) via the Insight app on smartphones for 6 months. Responses to these questions are used to personalize and tailor MAP treatment to each participant.

#### Assessment of Effectiveness

The pool of participants for this assessment is enrollees in AA-SH and AA-MAP. At 6 months post enrollment, a local research team member (not associated with ART clinics) will call the enrollees to assess smoking status. Participants who consented to being contacted after 6 months at enrollment and are reachable within 30 days of the target assessment date will undergo an informed consent process conducted by the research staff. Participants who provide written informed consent will be asked to complete a 30-minute survey in person at the ART clinic or via a phone or video call. Data will be collected using the REDCap mobile app on a tablet [63,64].

The total implementation period will be up to 36 months (enrollment will stop at 30 months and the last enrolled participant will be assessed at 36 months). We will assess up to 1200 treatment enrollees at 6 months. In our previous and current Lao and Cambodian studies, retention was 98% at 3 months and 90% at 6 months. Conservatively assuming only 60% (1863/3105) of AA-SH and AA-MAP enrollees can be contacted and agree to participate, there will be at least 1728 male and 135 female enrollees available to complete the 6-month assessment. Thus, a sample of 1200 enrollees is highly feasible. Given the hypothesized greater reach of AA-SH (90%) over AA-MAP (70%), we anticipate around 700 in the AA-SH group and around 500 in the AA-MAP group.

#### Primary Outcome

The primary outcome to evaluate effectiveness is smoking status at 6 months post enrollment. Participants who self-report 7-day point prevalence abstinence will be seen in person at the clinic within 30 days post survey for biochemical verification. We will try to arrange this visit to coincide with participants’ regular or prescheduled clinic visit for ART or other services. At the in-clinic appointment, they will be asked again, “Did you smoke cigarettes in the past 7 days?” before breath carbon monoxide will be measured by Smokerlyzer [65]. Abstinence is defined as having expired carbon monoxide <6 ppm [66].

#### Other Outcomes

We will also estimate the impact of AA-SH and AA-MAP. Impact, the multiplicative effect of reach and effectiveness, is crucial in evaluating health intervention implementation. An intervention may have a broad reach but limited effectiveness, or have strong effectiveness but limited reach, and hence ultimately may have limited public health impact [67,68]. Other measures of aim 1 are listed in Table 2. Paper and District Health Information Software 2 [69] medical records will be used to collect patients’ clinical information (with their consent), such as most recent CD4 lymphocyte counts, viral loads, medication, tuberculosis, and other comorbidities.

**Table 2.** Study assessments.

Variable type and operationalization measure	Assessment type and time
Patients: demographic and medical information	
Demographics; health literacy [70]	PA6 and medical record extraction
Alcohol and drug use [71,72]	PA6 and medical record extraction
CD4 lymphocyte count, viral load, tuberculosis, comorbidities	PA6 and medical record extraction
Nicotine dependence—Heaviness of Smoking Index [73]	PA6 and medical record extraction
Self-report measures of antiretroviral therapy adherence [74]	PA6 and medical record extraction
Patients: phase-based model mechanisms	
Wisconsin Smoking Withdrawal Scale [75]	Weekly EMA^a^^,^^b^
Reasons for quitting [76], number of quit attempts	PA6^c^
Nicotine replacement therapy use	PA6 and EMA
K10^d^ [77]	Weekly EMA^b^
PANAS^e^ [78]	Weekly EMA^b^
Multidimensional Scale of Perceived Social Support [79]	PA6
Self-efficacy (related to smoking cessation) [80]	Weekly EMA^b^
Patients: adherence to treatment app	
Duration of phone on/off; number of messages/images delivered and viewed; number of weekly EMAs opened and completed; data syncing frequencies	Digital logs in the Insight platform
Patients: primary outcomes of aim 1	
Reach, [81] the proportion of people with HIV who smoke and enroll	REDCap, PA6, and in-person expired carbon monoxide assessment
Effectiveness, [81] the proportion of enrolled participants who achieve biochemically confirmed point prevalence abstinence (expired carbon monoxide <6 ppm [66]) at the assessment at 6 months	REDCap, PA6, and in-person expired carbon monoxide assessmentREDCap, PA6, and in-person expired carbon monoxide assessment
Impact [67,68] = reach × effectiveness	REDCap, PA6, and in-person expired carbon monoxide assessment
Intervention characteristics	
Intervention Appropriateness, Acceptability, and Ease-of-Implementation Measures [82]	Staff surveys
Characteristics of the interventions that may facilitate or impede the clinical and implementation outcomes	PA6 and clinic staff IDIs
Clinics: PRISM^f^ mechanisms	
Organizational characteristics, patient population size, number of staff, sources of funding	KIIs^g^ with leaders
Adoption, proportion of staff implementing the interventions, characteristics of participating and nonparticipating staff	KIIs, staff surveys
Implementation, fidelity to the implementation strategies (proportion of clinic’s patients and visits asked for smoking status), cointervention, documentation of unplanned adaptations (FRAME-IS) [83]	REDCap, KII
Sustainability, NoMAD^h^ [84], facilitators/barriers to intervention sustainment	Staff survey, IDIs^i^
External factors	
Policies, resources, guidelines, and incentives [61] that affect the implementation and sustainability	KIIs
Economic evaluation	
Resources and costs of AA-SH^j^/AA-MAP^k^ implementation, cost per quitter for each implementation strategy [57], cost-effectiveness of AA-MAP vs AA-SH	(See the description in Aim 3 section)
EQ-5D-5L (for health utilities) [85]	PA6

a EMA: ecological momentary assessment.

b Brief versions of the scales, delivered weekly during the 6-month smoking cessation treatment period as an ecological momentary assessment via our app on a smartphone*.*

c PA6: patient assessment at 6 months.

d K10: Kessler Psychological Distress Scale.

e PANAS: Positive and Negative Affect Schedule.

f PRISM: Practical, Robust Implementation and Sustainability Model.

g KII: key informant interview.

h NoMAD: Normalization Measure Development.

i IDI: in-depth interview.

j AA-SH: Ask-Advice-Connect–self-help material.

k AA-MAP: Ask-Advice-Connect–mHealth-based automated treatment program.

#### Assessment of Multilevel Implementation Outcomes

##### Clinic Staff Survey (Quantitative)

At the end of this trial, all ART clinic staff will be invited to participate in a 30-minute self-administered survey delivered by REDCap on a tablet. Those who are eligible and provide written informed consent will participate. The survey will collect data regarding clinic staff characteristics, perceptions about the interventions, normalization, implementation, and sustainment facilitators or barriers (with structured questions), and other relevant variables listed in Table 2. Measures will be translated into Lao, mirroring the WHO’s recommended methodology [86,87] that we used for the measures and intervention messages in our R21/R33 study.

##### IDI (Qualitative)

All clinic staff who are assigned to and/or directly involved in implementing AA-SH or AA-MAP (eg, counselors, nurses, or physicians) will be invited to participate in a face-to-face IDI at the end of this trial. We expect that 3‐5 clinic staff members at each clinic will be assigned to this implementation role; thus, we target a sample size of 25‐40 for 8 clinics. This sample size is sufficient for reaching saturation [88], but additional staff may be recruited if saturation is not achieved. The IDIs will be conducted in Lao language by Lao research staff who have at least a master’s degree in health sciences, who have previous experience with qualitative interviews (in our R21 or other studies), and who will be additionally trained for conducting IDIs in this study. The IDI will include open-ended questions to explore implementation and sustainment facilitators or barriers, and areas for improvement.

##### KII (Qualitative)

At the end of this trial, we will conduct face-to-face interviews with up to 20 key informants, who are ART clinic directors or leaders, key stakeholders of the hospitals or provincial health departments, CHAS technical officers and authorities, NTCC stakeholders, and Lao MOH stakeholders. The intent of the KII is to explore external factors and context (Table 2) that may affect the intervention strategies’ implementation, sustainment, and future dissemination. Other aspects of the KII are similar to those of the IDI.

### Data Analysis Plan

#### Aim 1: Evaluate the Reach and Effectiveness of AA-MAP Versus AA-SH

The primary outcome for reach is the percentage of smokers willing to make a quit attempt who accept the self-help guide (in the AA-SH condition) or sign up for the MAP (in the AA-MAP condition). We hypothesized that reach would be greater for AA-SH (90%) than for AA-MAP (70%). Generalized linear mixed models (GLMMs) will be applied to evaluate the difference between AA-SH and AA-MAP. In addition to condition as a fixed effect, the primary model will include clinic as a random effect, given the potential for differences in acceptance and downloading due to the clinic. Expected denominators are at least 3000 (2880 men and 225 women) for both AA-SH and AA-MAP. With α=.05, a 2-sided test, an intracluster correlation coefficient of 0.22 or less, and the hypothesized enrollment rates of 90% for AA-SH and 70% for AA-MAP, power is greater than 0.99. Furthermore, power will be greater than 0.80 for enrollment rates of AA-MAP that are at least 5% lower than AA-SH with AA-SH rates of 60% or higher. Additional analyses will explore factors associated with reach within the AA-SH and AA-MAP conditions to guide future adaptation of either intervention.

The primary outcome for effectiveness is the biochemically verified 7-day point prevalence abstinence at the 6-month assessment. Those who fail to complete biochemical verification will be imputed as smoking. Based on our previous studies with US or Cambodian people with HIV, we predict abstinence rates at 6 months will be 8% (4/50) for AA-SH and 16% (8/50) for AA-MAP (odds ratio [OR] 2.20). This estimate of abstinence in the MAP group is based on the high end-of-treatment abstinence (40%) observed in our pilot RCT with Cambodian people with HIV, along with the many observations of high relapse rates at long-term follow-up among people with HIV [89,90]. GLMM will be used to evaluate the difference between AA-MAP and AA-SH in abstinence rates at 6 months post enrollment. Condition will be entered as a fixed effect in the model, along with clinic as a random effect. Any smoking-related or demographic variable found to differ with *P*<.10 across the conditions will also be included in the model (eg, biological sex). With an expected 700 participants in AA-SH and 500 in AA-MAP, α=.05, a 2-sided test, and an intracluster correlation coefficient of 0.10, power is greater than 0.98 to detect the predicted difference between the intervention groups. Furthermore, power will be greater than 0.80 for (1) abstinence rates equal to 8% for AA-SH and 13% or higher for AA-MAP (OR 1.72) with the same sample sizes, or (2) for abstinence rates equal to 8% and 16% (OR 2.20) with sample sizes of 300 for AA-SH and 250 for AA-MAP. Exploratory analyses will be performed. Differences between those who are abstinent and those who are not abstinent will be evaluated using *t* test and a chi-square test. GLMMs or linear mixed models will be used to examine other smoking-related outcomes, such as self-reported continuous abstinence, prolonged abstinence, and number of quit attempts. Additional analyses will explore factors associated with effectiveness within the AA-SH and AA-MAP conditions to guide future adaptation of either intervention.

#### Aim 2: Mixed Methods Data Analysis to Evaluate Other Multilevel Implementation Outcomes of AA-SH or AA-MAP

For quantitative data (staff survey and REDCap data), we will use descriptive statistics to characterize clinics, staff, and implementation outcomes. We will also use appropriate statistical methods (eg, chi-squared test or logistic regression) to examine the associations between clinic or staff characteristics and implementation outcomes. All qualitative interviews (IDIs and KIIs) will be recorded, transcribed verbatim, and then translated into English for analysis using thematic analysis [91]. We will use a hybrid approach [92] for the thematic analyses by developing deductive codes [93] based on PBM and PRISM constructs and inductive codes that emerge from the data [94]. At least 2 research team members will code all transcripts with the aid of MAXQDA [95]. Discrepancies will be discussed to achieve intercoder agreement. We will ask a small group of interview participants (n=5) to review the themes to ensure that our interpretation of the data is consistent with their experiences (ie, member checking) [96]. We will develop a mixed methods data inventory [97] and a matrix to examine quantitative and qualitative data jointly. We will use the integration strategies of merging and corroborating with the intent to explore what might have affected implementation outcomes [97,98]. We will also use data reduction to identify data that were primarily useful for this analytical focus (ie, qualitative and quantitative data of the same themes or areas but may converge or diverge). Mixed methods linked data, results, and meta-inferences will be presented in visual joint displays [98], similar to our previous publication [34,99].

#### Aim 3: Conduct a Comprehensive Evaluation of the Resource and Cost Associated with AA-SH or AA-MAP

Information on the resources needed, total costs, and cost-effectiveness estimates of AA-SH and AA-MAP are critical for partnering with Lao government agencies to implement and scale up the interventions at ART and other clinics throughout Laos. Therefore, to inform decision-making of public sector stakeholders, we will conduct an analysis of the resources needed to widely implement the interventions, and the potential gain from investing those resources from the perspective of the government service providers.

Our resource use and effectiveness methodology will follow current best-practice guidelines [100-102]. In keeping with the PRISM framework and the goal to inform the Lao government of resource use, costs, and gains from AA-SH or AA-MAP implementation, the analysis of resources will be from the perspective of government payers, initially not including related expenses such as patient out-of-pocket costs. In addition, we will use the gold-standard methodology of measuring resource use and assigning generalized prices to each type of resource used [103-105], resulting in “costs” that are comparable across different intervention sites and patient populations. We have successfully used this methodology in a variety of previous studies [106-111].

We will develop a project-specific version of the cost assessment methodology (CAM) template as we did in previous studies [106-111]. The CAM template streamlines the process of identifying and collecting data on the resource usage and costs of community and clinical interventions. Advantages of the CAM approach are that the cost results are not tied to the specific location of the intervention, which may have higher or lower general costs (eg, city vs rural), and it does not “miss” intervention resources—and thus costs—that may have been donated or subsidized for this study. Consequently, the data collected as part of aim 3 can be used to predict the costs for implementing the interventions in new areas and/or countrywide in Laos. The project-specific data for the CAM template will be informed by steps 1‐3 listed below, and calculations in step 4 will use these data. All data delineation and collection will be completed in close consultation with all of the investigators, particularly with the Lao coinvestigators.

#### CAM Approach

(1) Identify the individual components and phases of the interventions, and the resources needed to conduct each component. Examples of resource categories include personnel effort (eg, clinic staff and the national MAP core), material items (eg, self-help guides), and MAP infrastructure (eg, data storage). (2) As closely as possible, track the number of “units” of each type of resource that are used over the course of the implementation of each of the 2 intervention strategies (eg, hours of personnel time, number of self-help guides delivered, and MAP server rentals). Where precise or even approximate tracking of specific inputs to the intervention is not possible, the research team will develop estimates, including a range that can be used for sensitivity analyses. (3) Identify a generalized “price” per unit for each type of resource to determine a total cost for that resource by multiplying price by the total number of resources. These prices will be drawn from Lao national or representative sources. For example, the price per hour for the counselors or nurses will be collected from the Lao MOH payroll record. (4) Calculate total units and costs of the different resources used; total costs per intervention strategy; cost per patient participant in each RCT arm; and cost-effectiveness of the AA-MAP relative to AA-SH using the abstinence rate in each arm at 6 months.

## Results

The study has been funded since August 2024. We have obtained all of the necessary IRB and regulatory approvals for this study. We have adapted the content of our MAP (previously developed for the Lao general patient population) to ensure that the content is comprehensible and relevant to smokers living with HIV in Laos. We have also created additional smoking cessation and wellness messages (eg, harms of smoking related to HIV progression and treatment, reducing stress including HIV-related stress, or ART adherence while quitting smoking) to ensure that the treatment content is tailored to smokers with HIV and their comorbidities. We completed working with the Lao team to translate and back-translate the treatment content and other study materials from English to Lao. As of September 2026, we have launched the implementation of AA-SH and AA-MAP at four ART clinics. We plan to expand the implementation at the other ART clinics by December 2026. Data in Insight and REDCap will be monitored to ensure protocol fidelity. Booster training sessions for ART clinic staff will be used to obtain feedback and address emerging issues concerning implementation.

The multiple principal investigators have met with the selected ART clinics, explained this study, and discussed an implementation plan and potential challenges. The clinic leaders and staff provided several pieces of information that will help facilitate this project’s implementation, such as the fact that almost all ART patients have a mobile phone number (for clinic staff to contact them for their ART services) and 80% use a smartphone. Most challenges and concerns were addressed with our proposed approach; for example, older or low-income patients without a smartphone (solution: the self-help guide will be available to all clinics at the end of this trial), patients changing clinics for ART may not be reachable for follow-up (solution: CHAS has their contact information in the database), patient confidentiality (solution: we explained our plan for human participants’ protection), or potential staff’s heavy workload (solution: we explained the brief Ask-Advice-Connect procedure). At the end of these meetings, all the selected clinics expressed their commitment to implementing the proposed project and provided official letters of support. Three leaders of regional or national hospitals that house the ART clinics expressed their high enthusiasm to implement the proposed cessation strategies for all patients (not just ART patients) after the completion of this project.

Given the ART clinics’ commitment, we expect that all selected clinics will fully participate. However, if a clinic cannot participate, we and CHAS will select an alternative clinic. Additionally, because all ART clinics are under CHAS management and have performance goals and incentives (eg, yearly recognitions or awards from the Lao MOH), we expect no attrition. To minimize the risk of attrition further, we will have monthly video calls and quarterly in-person meetings with organizational leaders and compensate the clinics for costs and resources used for research purposes (eg, data collection and reporting). ART clinic staff turnover rate is low in Laos because all staff are governmental employees with a tenure-track or tenured mechanism. However, if a staff change occurs, newly hired staff will be trained on the AA-SH or AA-MAP implementation by using the recorded videos and by other current clinic staff.

## Discussion

### Anticipated Findings

Cigarette smoking is among the leading causes of cancer morbidity and mortality among people with HIV worldwide. This issue is even more striking in Laos due to a high smoking prevalence in people with HIV (men 61%‐80%, women 3%‐10%) and a lack of cessation treatment resources. Available data (ie, high ART coverage, evidence for the effectiveness of the proposed cessation strategies, and good telecommunication infrastructure) suggest that our proposed MAP intervention is highly feasible, scalable, affordable, and potentially sustainable.

The proposed 3-step Ask-Advice-Connect approach in this project has demonstrated its effectiveness and is recommended for wide adoption in routine HIV care settings in the United States [8]. A systematic review showed that brief advice from physicians (vs no advice or usual care) could significantly increase cessation (relative risk [RR] 1.66, 95% CI 1.42‐1.94) [112]. In the MPOWER framework, WHO also recommends that participating nations adopt effective measures to ensure adequate treatment for tobacco dependence, including integrating tobacco use screening and brief intervention into health care systems (article 14) [7]. Our approach, which involves delivering Ask-Advice-Connect to patients during regular visits to the ART clinics and connecting them to our evidence-based smoking cessation interventions, represents an ideal and highly feasible way to address this public health problem.

Our proposed tobacco treatment strategies, that is, self-help print materials and mHealth-based text messages, are also evidence-based and effective, independent of pharmacotherapies. A meta-analysis showed that standard, nontailored self-help materials had a moderate effect on smoking cessation when compared with no intervention (RR 1.19, 95% CI 1.03‐1.37) [113]. Furthermore, the US Surgeon General report states that “the evidence is sufficient to infer that SMSs about cessation are independently effective in increasing smoking cessation, particularly if they are interactive or tailored to individuals” [36]. Mobile phone text messaging is effective (pooled RR from a systematic review, 1.67‐1.83) [35] and cost-effective [37,40], and has been endorsed or used by WHO and other international organizations in their global tobacco control efforts [38,39]. Given the accumulated evidence base for advanced smartphone-delivered interventions (ie, apps and notifications), many agencies, such as the US NCI, have made scientifically supported apps available for public use [114]. WHO also recommends and provides a guide for low- and middle-income countries (LMICs) to adopt mHealth for smoking cessation [57]. However, the real-world impact of these interventions in LMIC settings is not well known. Nontailored self-help print materials can be implemented with minimal effort and resources but are less effective, while an interactive tailored mHealth intervention is more effective but may have lower reach. Our project will contribute to filling this knowledge gap.

Our approach is novel in many aspects. First, this study will be the first to shift the tobacco treatment paradigm and to change clinical practice and health care delivery (ie, to include smoking cessation support) in Laos. Second, the completion of our project aims will inform the LMIC tobacco control community about the effectiveness of a low-effort clinic-based Ask-Advice-Connect approach combined with health care providers’ authoritative voices and low-cost cessation interventions (eg, self-help guides or MAP). This contributing evidence has the potential to shift national LMIC tobacco treatment strategies (eg, moving from population-based to clinic-based for improved reach, or moving from a high-cost intensive counseling approach to an mHealth-based low-cost interactive and tailored program). Third, if the clinic-based approach is impactful for smoking cessation, the empirically based Ask-Advice-Connect approach, which is novel in Laos and in many LMICs, can be adapted and used for the future delivery of other cancer prevention services both to people with HIV and to other at-risk patient populations. Fourth, our approach to planning this project is novel. Lao national and local stakeholders have actively engaged in several in-person and video-call meetings to discuss all major aspects of this project and consider everyone’s interests (eg, CHAS selecting diverse study sites to evaluate the potential wide reach of the interventions, local hospital leaders’ wish to scale the interventions beyond just ART patients, and NTCC’s interest in the costs of implementing the mHealth treatment program nationwide). With these stakeholders’ buy-in, the implementation of this project is very likely to succeed. Fifth, our Insight platform is innovative in how it manages and delivers automatic, interactive, tailored smoking cessation content (text messages in the form of notifications plus images and videos). The versatile Insight platform enables intervention designers to launch secure technology-based assessments, track momentary changes in key variables in near real-time, and use this information to drive the just-in-time adaptive interventions. Insight allows our mHealth treatment to function autonomously and minimizes human involvement, making the approach very affordable for large-scale implementation in LMICs.

This study has some limitations. First, the parallel cluster RCT design (ie, without randomization at the individual level) may limit the statistical power for some analyses. However, because Ask-Advice-Connect is performed by the clinic staff, an individual RCT with patient randomization is not appropriate, and a cluster RCT is the best design for this hybrid type-2 pragmatic effectiveness-implementation study. Second, we are implementing the Ask-Advice-Connect approach to screen for cigarette smokers and not other forms of tobacco use (eg, vaping or tobacco chewing). However, >95% of tobacco users in Laos are cigarette smokers [3,4], and Laos currently bans all forms of e-cigarettes [3,5]. Thus, screening for other rare forms of tobacco use creates a burden for clinic staff, making this approach less cost-effective. Third, we considered the use of medications to aid smoking cessation. However, no prescription medications for tobacco use treatment are available in Laos. Additionally, because NRT is not covered by health insurance in Laos, providing NRT would limit the real-world implementation and sustainability of this study. Thus, we will provide information about NRT, such as nicotine patches and lozenges that participants can buy online or over the counter. Finally, while the use of Insight and smartphones may exclude some very low-income people with HIV who smoke, it is a more effective treatment approach that potentially maximizes the impact.

### Conclusions

Our proposed research project addresses the critical need for implementing evidence-based smoking cessation strategies for people with HIV in Laos. The US and Lao investigators co-designed this project with a vision that, if the proposed implementation strategies are found to have great impact, they will be broadly implemented for other patient populations in Lao hospitals in the future. The successful completion of this project will contribute important actionable inputs that will inform the influential Lao NTCC and MOH to implement the strategies in diverse hospital settings in future large-scale hybrid type II/III trials. Ultimately, our course of research has the potential to transform health care delivery and to contribute to reducing tobacco-related morbidities and mortalities in Laos.

## Supplementary material

10.2196/100518 Peer Review Report 1 Peer review report by the National Cancer Institute (NCI) of the United States National Institutes of Health.

## Abbreviations

AA-MAP: Ask-Advice-Connect–mHealth-based automated treatment program
AA-SH: Ask-Advice-Connect–self-help material
ART: antiretroviral therapy
CAM: cost assessment methodology
CHAS: Center for HIV/AIDS and STIs of Laos
CONSORT: Consolidated Standards of Reporting Trials
GLMM: Generalized linear mixed models
IDI: in-depth interview
IRB: Institutional Review Board
KII: key informant interview
LMIC: low- and middle-income country
MAP: mHealth-based automated treatment program
mHealth: mobile health
MOH: Ministry of Health
MPOWER: monitor tobacco use and prevention policies; protect people from tobacco smoke; offer help to quit tobacco use; warn about the dangers of tobacco; enforce bans on tobacco advertising, promotion, and sponsorship; and raise taxes on tobacco
NCI: National Cancer Institute
NCLE: National Center for Laboratory and Epidemiology
NRT: nicotine replacement therapy
NTCC: National Tobacco Control Committee
OR: odds ratio
PBM: phase-based model
PRISM: Practical, Robust Implementation and Sustainability Model
RCT: randomized controlled trial
RE-AIM: Reach, Effectiveness, Adoption, Implementation, and Maintenance
RR: relative risk
SPIRIT: Standard Protocol Items: Recommendations for Interventional Trials
WHO: World Health Organization

## References

[1] (2016). The economics of tobacco and tobacco control. national cancer institute tobacco control monograph 21. https://cancercontrol.cancer.gov/brp/tcrb/monographs/monograph-21.

[2] Population. Lao Statistics Bureau.

[3] (2021). WHO report on the global tobacco epidemic 2021 - country profile - lao people’s democratic republic. https://cdn.who.int/media/docs/default-source/country-profiles/tobacco/who_rgte_2021_lao_people-s_democratic_republic.pdf?sfvrsn=aa9587d0_5&download=true.

[4] Xangsayarath P, Douangvichith D, Siengsounthone L, Phandouangsy K, Tran LTH, Bui TC (2019). Tobacco use in Lao People’s Democratic Republic: results from the 2015 National Adult Tobacco Survey. Tob Prev Cessation.

[5] Tan YL, Dorotheo U (2021). Southeast Asia Tobacco Control Alliance (SEATCA).

[6] MPOWER. World Health Organization.

[7] (2023). WHO report on the global tobacco epidemic, 2023: protect people from tobacco smoke. https://www.who.int/publications/i/item/9789240077164.

[8] Reddy KP, Kruse GR, Lee S, Shuter J, Rigotti NA (2022). Tobacco use and treatment of tobacco dependence among people with human immunodeficiency virus: a practical guide for clinicians. Clin Infect Dis.

[9] Shirley DK, Kaner RJ, Glesby MJ (2013). Effects of smoking on non-AIDS-related morbidity in HIV-infected patients. Clin Infect Dis.

[10] Nahvi S, Cooperman NA (2009). Review: the need for smoking cessation among HIV-positive smokers. AIDS Educ Prev.

[11] Rahmanian S, Wewers ME, Koletar S, Reynolds N, Ferketich A, Diaz P (2011). Cigarette smoking in the HIV-infected population. Proc Am Thorac Soc.

[12] Reynolds NR (2009). Cigarette smoking and HIV: more evidence for action. AIDS Educ Prev.

[13] Lifson AR, Neuhaus J, Arribas JR (2010). Smoking-related health risks among persons with HIV in the strategies for management of antiretroviral therapy clinical trial. Am J Public Health.

[14] Helleberg M, May MT, Ingle SM (2015). Smoking and life expectancy among HIV-infected individuals on antiretroviral therapy in Europe and North America. AIDS.

[15] Ashare RL, Bernstein SL, Schnoll R (2021). The United States National Cancer Institute’s coordinated research effort on tobacco use as a major cause of morbidity and mortality among people with HIV. Nicotine Tob Res.

[16] Chaturvedi AK, Pfeiffer RM, Chang L, Goedert JJ, Biggar RJ, Engels EA (2007). Elevated risk of lung cancer among people with AIDS. AIDS.

[17] Clifford GM, Polesel J, Rickenbach M (2005). Cancer risk in the Swiss HIV Cohort Study: associations with immunodeficiency, smoking, and highly active antiretroviral therapy. J Natl Cancer Inst.

[18] Engels EA, Brock MV, Chen J, Hooker CM, Gillison M, Moore RD (2006). Elevated incidence of lung cancer among HIV-infected individuals. J Clin Oncol.

[19] Kirk GD, Merlo C, O’Driscoll P (2007). HIV infection is associated with an increased risk for lung cancer, independent of smoking. Clin Infect Dis.

[20] Sigel K, Wisnivesky J, Gordon K (2012). HIV as an independent risk factor for incident lung cancer. AIDS.

[21] Feldman JG, Minkoff H, Schneider MF (2006). Association of cigarette smoking with HIV prognosis among women in the HAART era: a report from the women’s interagency HIV study. Am J Public Health.

[22] Ale BM, Amahowe F, Nganda MM (2021). Global burden of active smoking among people living with HIV on antiretroviral therapy: a systematic review and meta-analysis. Infect Dis Poverty.

[23] Crothers K, Griffith TA, McGinnis KA (2005). The impact of cigarette smoking on mortality, quality of life, and comorbid illness among HIV-positive veterans. J Gen Intern Med.

[24] Helleberg M, Afzal S, Kronborg G (2013). Mortality attributable to smoking among HIV-1-infected individuals: a nationwide, population-based cohort study. Clin Infect Dis.

[25] Lindayani L, Yeh CY, Ko WC, Ko NY (2020). High smoking prevalence among HIV-positive individuals: a systematic review and meta-analysis. J Subst Use.

[26] Bui TC, Piñeiro B, Vidrine DJ, Wetter DW, Frank-Pearce SG, Vidrine JI (2020). Quitline treatment enrollment and cessation outcomes among smokers linked with treatment via Ask-Advise-Connect: comparisons among smokers with and without HIV. Nicotine Tob Res.

[27] Frazier EL, Sutton MY, Brooks JT, Shouse RL, Weiser J (2018). Trends in cigarette smoking among adults with HIV compared with the general adult population, United States - 2009-2014. Prev Med.

[28] Asfar T, Perez A, Shipman P (2021). National estimates of prevalence, time-trend, and correlates of smoking in US people living with HIV (NHANES 1999–2016). Nicotine Tob Res.

[29] Mdodo R, Frazier EL, Dube SR (2015). Cigarette smoking prevalence among adults with HIV compared with the general adult population in the United States: cross-sectional surveys. Ann Intern Med.

[30] Johnston PI, Wright SW, Orr M (2021). Worldwide relative smoking prevalence among people living with and without HIV. AIDS.

[31] Lao National Center for HIV/AIDS and STIs (CHAS) (2023). HIV/AIDS statistics.

[32] Bui TC, Hoogland CE, Chhea C (2023). Ending tobacco use through interactive tailored messaging for Cambodian people with HIV (Project EndIT): protocol for a randomized controlled trial. JMIR Res Protoc.

[33] Kulkarni S, Xangsayarath P, Xayavong D (2023). Abstract 63: mobile health technology for tailored tobacco cessation support in Lao People’s Democratic Republic: a pilot trial. Cancer Epidemiol, Biomarkers Prev.

[34] Bui TC, Sopheab H, Businelle MS (2022). Mobile-health intervention for smoking cessation among Cambodian people living with HIV: a mixed-methods pilot study. AIDS Care.

[35] Whittaker R, McRobbie H, Bullen C, Rodgers A, Gu Y (2016). Mobile phone-based interventions for smoking cessation. Cochrane Database Syst Rev.

[36] (2020). Smoking cessation: a report of the surgeon general. https://www.cdc.gov/tobacco-surgeon-general-reports/reports/2020-smoking-cessation/index.html#cdc_report_pub_study_section_2-full-report.

[37] Guerriero C, Cairns J, Roberts I, Rodgers A, Whittaker R, Free C (2013). The cost-effectiveness of smoking cessation support delivered by mobile phone text messaging: Txt2stop. Eur J Health Econ.

[38] Raw M, Mackay J, Reddy S (2016). Time to take tobacco dependence treatment seriously. Lancet.

[39] (2015). Mobile health for tobacco cessation (mTobaccoCessation). World Health Organization.

[40] West R, Raw M, McNeill A (2015). Health-care interventions to promote and assist tobacco cessation: a review of efficacy, effectiveness and affordability for use in national guideline development. Addiction.

[41] (2023). Measuring digital development facts and figures: focus on least developed countries. https://www.itu.int/itu-d/reports/statistics/facts-figures-for-ldc/.

[42] Statistical information service by theme - technology and communication. Lao Statistics Bureau.

[43] Vidrine JI, Shete S, Cao Y (2013). Ask-Advise-Connect: a new approach to smoking treatment delivery in health care settings. JAMA Intern Med.

[44] Vidrine JI, Shete S, Li Y (2013). The Ask-Advise-Connect approach for smokers in a safety net healthcare system: a group-randomized trial. Am J Prev Med.

[45] Feldstein AC, Glasgow RE (2008). A Practical, Robust Implementation and Sustainability Model (PRISM) for integrating research findings into practice. Jt Comm J Qual Patient Saf.

[46] Rabin BA, Cakici J, Golden CA, Estabrooks PA, Glasgow RE, Gaglio B (2022). A citation analysis and scoping systematic review of the operationalization of the Practical, Robust Implementation and Sustainability Model (PRISM). Implementation Sci.

[47] Frequently asked questions about PRISM. RE-AIM.

[48] Zwarenstein M, Treweek S, Gagnier JJ (2008). Improving the reporting of pragmatic trials: an extension of the CONSORT statement. BMJ.

[49] Butcher NJ, Monsour A, Mew EJ (2022). Guidelines for reporting outcomes in trial protocols: the SPIRIT-outcomes 2022 extension. JAMA.

[50] Baker TB, Mermelstein R, Collins LM (2011). New methods for tobacco dependence treatment research. Ann Behav Med.

[51] Piper ME, Schlam TR, Cook JW (2011). Tobacco withdrawal components and their relations with cessation success. Psychopharmacology (Berl).

[52] Piper ME, Schlam TR, Cook JW (2017). Toward precision smoking cessation treatment I: moderator results from a factorial experiment. Drug Alcohol Depend.

[53] Piper ME, Cook JW, Schlam TR (2017). Toward precision smoking cessation treatment II: proximal effects of smoking cessation intervention components on putative mechanisms of action. Drug Alcohol Depend.

[54] Piper ME, Fiore MC, Smith SS (2016). Identifying effective intervention components for smoking cessation: a factorial screening experiment. Addiction.

[55] Key facts on HIV - Lao PDR. AIDS Data Hub.

[56] (2023). The Mobile Health (mHealth) Shared Resource. OUHealth - Stephenson Cancer Center.

[57] (2015). Be healthy, be mobile: a handbook on how to implement mTobaccoCessation. https://www.who.int/publications/i/item/9789241549813.

[58] Baker TB, Collins LM, Mermelstein R (2016). Enhancing the effectiveness of smoking treatment research: conceptual bases and progress. Addiction.

[59] Bellg AJ, Borrelli B, Resnick B (2004). Enhancing treatment fidelity in health behavior change studies: best practices and recommendations from the NIH Behavior Change Consortium. Health Psychol.

[60] Carroll C, Patterson M, Wood S, Booth A, Rick J, Balain S (2007). A conceptual framework for implementation fidelity. Implement Sci.

[61] Glasgow RE, Harden SM, Gaglio B (2019). RE-AIM planning and evaluation framework: adapting to new science and practice with a 20-year review. Front Public Health.

[62] Creswell JW (2021). A Concise Introduction to Mixed Methods Research.

[63] Harris PA, Taylor R, Minor BL (2019). The REDCap consortium: building an international community of software platform partners. J Biomed Inf.

[64] Harris PA, Taylor R, Thielke R, Payne J, Gonzalez N, Conde JG (2009). Research electronic data capture (REDCap)--a metadata-driven methodology and workflow process for providing translational research informatics support. J Biomed Inf.

[65] Smokerlyzer. coVita.

[66] Benowitz NL, Iii PJ, Ahijevych K (2002). Biochemical verification of tobacco use and cessation. Nicotine Tob Res.

[67] Glasgow RE, Vogt TM, Boles SM (1999). Evaluating the public health impact of health promotion interventions: the RE-AIM framework. Am J Public Health.

[68] Rabin BA, Brownson RC, Haire-Joshu D, Kreuter MW, Weaver NL (2008). A glossary for dissemination and implementation research in health. J Public Health Manag Pract.

[69] University of Oslo. DHIS2.

[70] Chew LD, Bradley KA, Boyko EJ (2004). Brief questions to identify patients with inadequate health literacy. Fam Med.

[71] Screening Tools and Prevention. National Institute on Drug Abuse.

[72] (2010). The Alcohol, Smoking, and Substance Involvement Screening Test (ASSIST). World Health Organization.

[73] Heatherton TF, Kozlowski LT, Frecker RC, Fagerström KO (1991). The Fagerström Test for Nicotine Dependence: a revision of the Fagerström Tolerance Questionnaire. Br J Addict.

[74] Simoni JM, Kurth AE, Pearson CR, Pantalone DW, Merrill JO, Frick PA (2006). Self-report measures of antiretroviral therapy adherence: a review with recommendations for HIV research and clinical management. AIDS Behav.

[75] Welsch SK, Smith SS, Wetter DW, Jorenby DE, Fiore MC, Baker TB (1999). Development and validation of the Wisconsin Smoking Withdrawal Scale. Exp Clin Psychopharmacol.

[76] Curry SJ, Grothaus L, McBride C (1997). Reasons for quitting: intrinsic and extrinsic motivation for smoking cessation in a population-based sample of smokers. Addict Behav.

[77] (2018). Health translation - Kessler 10 (K10) assessment form. http://www.healthtranslations.vic.gov.au/bhcv2/bhcht.nsf/PresentDetail?Open&s=Kessler_10_(K10)_Assessment_Form.

[78] Watson D, Clark LA, Tellegen A (1988). Development and validation of brief measures of positive and negative affect: the PANAS scales. J Pers Soc Psychol.

[79] Zimet GD, Dahlem NW, Zimet SG, Farley GK (1988). The Multidimensional Scale of Perceived Social Support. J Pers Assess.

[80] Velicer WF, Diclemente CC, Rossi JS, Prochaska JO (1990). Relapse situations and self-efficacy: an integrative model. Addict Behav.

[81] What is RE-AIM?. RE-AIM.

[82] Weiner BJ, Lewis CC, Stanick C (2017). Psychometric assessment of three newly developed implementation outcome measures. Implement Sci.

[83] Miller CJ, Barnett ML, Baumann AA, Gutner CA, Wiltsey-Stirman S (2021). The FRAME-IS: a framework for documenting modifications to implementation strategies in healthcare. Implement Sci.

[84] Finch TL, Girling M, May CR (2018). Improving the normalization of complex interventions: part 2 - validation of the NoMAD instrument for assessing implementation work based on normalization process theory (NPT). BMC Med Res Methodol.

[85] Herdman M, Gudex C, Lloyd A (2011). Development and preliminary testing of the new five-level version of EQ-5D (EQ-5D-5L). Qual Life Res.

[86] Bullinger M, Alonso J, Apolone G (1998). Translating health status questionnaires and evaluating their quality: the IQOLA Project approach. J Clin Epidemiol.

[87] (2018). Research tools: process of translation and adaptation of instruments [archived version]. World Health Organization.

[88] Hennink M, Kaiser BN (2022). Sample sizes for saturation in qualitative research: a systematic review of empirical tests. Soc Sci Med.

[89] Gritz ER, Danysh HE, Fletcher FE (2013). Long-term outcomes of a cell phone-delivered intervention for smokers living with HIV/AIDS. Clin Infect Dis.

[90] Stanton CA, Kumar PN, Moadel AB (2020). A multicenter randomized controlled trial of intensive group therapy for tobacco treatment in HIV-infected cigarette smokers. J Acquired Immune Defic Syndr.

[91] Graneheim UH, Lundman B (2004). Qualitative content analysis in nursing research: concepts, procedures and measures to achieve trustworthiness. Nurse Educ Today.

[92] Fereday J, Muir-Cochrane E (2006). Demonstrating rigor using thematic analysis: a hybrid approach of inductive and deductive coding and theme development. Int J Qual Methods.

[93] Atkins L, Francis J, Islam R (2017). A guide to using the Theoretical Domains Framework of behaviour change to investigate implementation problems. Implement Sci.

[94] Hamilton AB, Finley EP (2019). Qualitative methods in implementation research: an introduction. Psychiatry Res.

[95] MAXQDA 2020.

[96] Creswell JW, Clark VLP (2017). Designing and Conducting Mixed Methods Research.

[97] Fetters MD (2019). The Mixed Methods Research Workbook: Activities for Designing, Implementing, and Publishing Projects.

[98] Guetterman TC, Fetters MD, Creswell JW (2015). Integrating quantitative and qualitative results in health science mixed methods research through joint displays. Ann Fam Med.

[99] Nguyen OT, Turner K, Charles D (2023). Implementing digital scribes to reduce electronic health record documentation burden among cancer care clinicians: a mixed-methods pilot study. JCO Clin Cancer Inf.

[100] Husereau D, Drummond M, Augustovski F (2022). Consolidated Health Economic Evaluation Reporting Standards 2022 (CHEERS 2022) statement: updated reporting guidance for health economic evaluations. MDM Policy Pract.

[101] Neumann PJ, Kim DD, Trikalinos TA (2018). Future directions for cost-effectiveness analyses in health and medicine. Med Decis Making.

[102] Sanders GD, Neumann PJ, Basu A (2016). Recommendations for conduct, methodological practices, and reporting of cost-effectiveness analyses: second panel on cost-effectiveness in health and medicine. JAMA.

[103] Russell LB, Gold MR, Siegel JE, Daniels N, Weinstein MC (1996). The role of cost-effectiveness analysis in health and medicine. JAMA.

[104] Weinstein MC, Siegel JE, Gold MR, Kamlet MS, Russell LB (1996). Recommendations of the panel on cost-effectiveness in health and medicine. JAMA.

[105] Dranove D, Dranove D (1996). Valuing Health Care Costs: Costs, Benefits and Effectiveness of Pharmaceuticals and Other Medical Technologies.

[106] Byrne MM, Koru-Sengul T, Zhao W, Weissfeld JL, Roberts MS (2010). Healthcare use after screening for lung cancer. Cancer.

[107] Byrne MM, Parker DF, Tannenbaum SL (2014). Cost of a community-based diabetic retinopathy screening program. Diabetes Care.

[108] Martinez U, Simmons VN, Sutton SK (2021). Targeted smoking cessation for dual users of combustible and electronic cigarettes: a randomised controlled trial. Lancet Public Health.

[109] Mason NT, Joshi VB, Adashek JJ (2023). Cost effectiveness of treatment sequences in advanced renal cell carcinoma. Eur Urol Oncol.

[110] Morgan RO, Byrne MM, Hughes RB (2008). Do secondary conditions explain the relationship between depression and health care cost in women with physical disabilities?. Arch Phys Med Rehabil.

[111] Simmons VN, Sutton SK, Medina-Ramirez P (2022). Self-help smoking cessation intervention for Spanish-speaking Hispanics/Latinxs in the United States: a randomized controlled trial. Cancer.

[112] Stead LF, Buitrago D, Preciado N, Sanchez G, Hartmann-Boyce J, Lancaster T (2013). Physician advice for smoking cessation. Cochrane Database Syst Rev.

[113] Livingstone-Banks J, Ordóñez-Mena JM, Hartmann-Boyce J (2019). Print-based self-help interventions for smoking cessation. Cochrane Database Syst Rev.

[114] smokefree.gov.

