# Supplementary material for: Implementing Sustainable Mobile Health Technology to Optimize a Smoking Cessation Program for Lao People With HIV (Project I-STOP): Protocol for a Hybrid Type-2 Pragmatic Effectiveness-Implementation Study
Source: JMIR Res Protoc. 2026 Oct 5;15:e100518. doi: 10.2196/100518 (PMC13638074; doi:10.2196/100518)
Supplement: Supplementary file 1 [file resprot-v15-e100518-s001.pdf]

SUMMARY STATEMENT

PROGRAM CONTACT:
James Alaro
240-276-6429
james.alaro@nih.gov

(Privileged Communication)

Release Date: 03/28/2024
Revised Date:

Application Number: 1U01CA294811-01

Principal Investigators (Listed Alphabetically):

BUI, THANH C. (Contact)
SANGXAYARATH, PHONEPADITH
VIDRINE, DAMON J.

Applicant Organization: UNIVERSITY OF OKLAHOMA HLTH SCIENCES CTR

Review Group: ZCA1 RPRB-M (M2)
National Cancer Institute Special Emphasis Panel
NCI Research Project-Cooperative Agreements (U01)
AIDS

Meeting Date: 03/13/2024
Council: MAY 2024
Requested Start: 07/01/2024

Opportunity Number: RFA-CA-23-033
PCC: AKGH

Project Title: Implementing Sustainable mobile health Technology to Optimize smoking cessation Program for Lao people with HIV (I-STOP)
SRG Action: Impact Score:25
Next Steps: Visit https://grants.nih.gov/grants/next_steps.htm
Human Subjects: 30-Human subjects involved - Certified, no SRG concerns
Animal Subjects: 10-No live vertebrate animals involved for competing appl.
Gender: 1A-Both genders, scientifically acceptable
Minority: 5A-Only foreign subjects, scientifically acceptable
Age: 3A-No children included, scientifically acceptable

Project Year	Direct Costs Requested	Estimated Total Cost
1	493,634	710,921
2	499,351	719,154
3	494,351	711,953
4	497,851	716,994
5	488,831	704,003
TOTAL	2,474,018	3,563,025

1U01CA294811-01 Bui, Thanh

RESUME AND SUMMARY OF DISCUSSION: This is a new U01 application in response to RFA-CA-23-033 from MPIs Dr. Thanh Bui at University of Oklahoma, Dr. Phonepadith Xangsayarath at the Ministry of Health of Lao PDR, and Dr. Damon Vidrine at Lee Moffitt Cancer Center. The overall goal of this study is to compare 2 smoking cessation implementation strategies in 8 antiretroviral therapy (ART) clinics among people living with HIV in the 5 most populous provinces/regions across Laos, using a hybrid type-2 pragmatic effectiveness-implementation study and a parallel cluster randomized trial design. Strengths of include excellent team of investigators with extensive experience, good mix of HIC and LMIC investigators, complimentary expertise that build on extensive work in PLHIV, and ongoing collaboration with key stakeholders. Also, there is great preliminary data, comprehensive assessment of resource use and cost effectiveness with rigorous strategy and robust models, the use of potentially scalable technology is innovative, and there is adequate data management plan and personalized interventions. Additionally, the involvement of Lao Ministry of Health, the cost-effective analysis addresses low cost of intervention advocate for nationwide implementation. Weaknesses noted include unclear presentation of morbidity and cancer risk in Lao PDR, potential selection bias against those who do not own a smart phone, and research infrastructure not well described. Significant previous efforts have been done in Laos but funding was a big problem and it is unclear how this project will build on this. Overall, this Project has the potential for high impact in the field.

DESCRIPTION (provided by applicant): Tobacco use remains the leading modifiable risk factor for preventing cancer globally, particularly among people with HIV (PWH). In Lao People's Democratic Republic (Laos), 61%–80% of male PWH and 3%–10% of female PWH smoke cigarettes. PWH who smoke in Laos currently have no theoretically and empirically based smoking cessation support. Thus, implementing sustainable and evidence-based smoking cessation interventions for PWH in Laos is critically needed. There is substantial evidence that mobile health (mHealth)- based smoking cessation interventions are effective, cost-effective, and affordable. We developed a scalable and affordable mHealth-based automated treatment program (MAP) to support Lao and Cambodian smokers to quit smoking. The MAP involves interactive, tailored, personalized content (text messages, photos, and videos) delivered via a smartphone app. Along with effective cessation treatments, it is imperative to implement procedures to identify patients who smoke and to facilitate connections to treatment. One such approach pioneered by our team is Ask-Advise-Connect (AAC, asking patients about smoking at every visit, briefly advising those who smoke to quit, and connecting them to treatment), which showed great impact in our US studies. The purpose of this application is to compare 2 smoking cessation implementation strategies in 8 antiretroviral therapy (ART) clinics in the 5 most populous provinces/regions across Laos, using a hybrid type-2 pragmatic effectiveness-implementation study and a parallel cluster randomized trial design. Specifically, we will compare an AAC approach paired with our previously developed MAP (AA-MAP) to an AAC approach paired with less resource intensive printed self-help material (AA-SH). To guide our implementation work, we use the Practical, Robust Implementation and Sustainability Model (PRISM), which expands the RE-AIM (Reach, Effectiveness, Adoption, Implementation, and Maintenance) framework to include more key, multilevel contextual factors relevant to program implementation. Aim 1 is to evaluate the reach and effectiveness of AA-MAP versus AA-SH. Reach is the proportion of PWH who smoke and are willing to make a quit attempt that enroll in treatment. Effectiveness is the proportion of enrolled participants (n=1,200) who achieve biochemically confirmed point prevalence abstinence 6 months after enrollment. We hypothesize that compared with AA-SH, AA-MAP will have a lower reach but will be more effective. We will also estimate the real-world impact (impact = reach × effectiveness) of each intervention. Aim 2 is to evaluate other multi-level implementation outcomes (e.g., adoption, implementation fidelity, and sustainability) of AA-MAP and AA-SH in the ART clinic setting using mixed methods. Aim 3 is to conduct a comprehensive assessment of the resource use and costs of implementing AA-MAP and AA-SH and calculate the absolute and relative cost effectiveness of the 2 intervention strategies. The project has the potential to

transform HIV care delivery throughout the country and to reduce tobacco-related cancers and other morbidities.

PUBLIC HEALTH RELEVANCE: This project is a collaborative initiative between US researchers and Lao Ministry of Health officials to compare 2 smoking cessation implementation strategies for people with HIV who smoke in 8 antiretroviral therapy clinics in Laos. The proposed research is relevant to public health because it is ultimately expected to advance understanding of real-world impact of smoking cessation approaches in low- and middle-income settings. Findings from this project will contribute evidence that has the potential to shift national tobacco treatment strategies in Laos and in other low- and middle-income countries and subsequently reduce tobacco-induced cancer morbidity and mortality.

CRITIQUE: The written critiques of individual reviewers are provided in essentially unedited form in this section. Please note that critiques and criteria scores, prepared prior to the review meeting, may not have been revised following discussions at the meeting. The "Resume and Summary of Discussion" section summarizes the final opinions of the review committee.

CRITIQUE 1

Significance:	5
Investigator(s):	2
Innovation:	3
Approach:	4
Environment:	4

Overall Impact: The proposed work comes from a multidisciplinary team of investigators with complimentary expertise building on their preliminary extensive work in mHealth and smoking cessation interventions including in people living with HIV. There is a clear institutional relationship between the US investigators and investigators from Lao People's Democratic Republic (Laos). The proposed project tackles a public health problem in Laos and builds on the investigators' prior work. However, there is insufficient data of cancer epidemiology for Laos. Nevertheless, the investigators have used a rigorous research strategy to achieve specific aims. Though there are some limitations in the mHealth-based automated treatment program in that it requires use of smartphones which can deter scale up of this specific intervention, the project has a potential to create a powerful influence on the smoking cessation programs incorporating mHealth.

1. Significance:
Strengths

- The proposal tackles a public health problem in Laos. Smoking, with its associated increased risk for cancer globally, is highly prevalent in Laos and more common among people with HIV than those from the general population.
- Though the regulatory framework for tobacco control measures exists including the establishment of the National Tobacco Control Committee of Laos, there is clear lack of treatment or smoking cessation support for the at-risk population particularly people with HIV.
- The proposed mHealth technologic interventions have been investigated by the applicants before, and the current proposed work builds on their previous collaboration between the researchers at the University of Oklahoma Health Sciences Center (OUHSC), Moffitt Cancer Center, Lao NTCC, and Lao Ministry of Health (MOH).
- Evaluating effective smoking cessation interventions that are potentially scalable is a unique strength of this application.
- The partnership of the investigators is an established strength that will provide a good platform for scale up.

Weaknesses

- There have been previous efforts to establish smoking cessation programs e.g. a Quitline with telephone counseling at national Mahosot Hospital which is a study site for this proposed work, but funding was a huge limitation. The investigators have not clearly described how they will address the barriers to prior efforts and ensure successful scale up though they have highlighted the direct involvement of Lao MOH and other governmental agencies.
- The investigators have not adequately included any background data of smoking and cancer epidemiology in Lao People's Democratic Republic (Laos).

2. Investigator(s):
Strengths

- The project has an excellent team of investigators with complimentary expertise including in mHealth technology, implementation science, smoking cessation interventions, economic evaluation, to see out the implementation of the proposed work.
- The team has extensive experience developing and evaluating mHealth based intervention including smoking cessation among people with HIV and in low-and-middle income settings.
- There is clear evidence of ongoing collaboration between the US investigators and the Lao People's Democratic Republic investigators including involvement of high-level policy makers including directors, Vice minister of Health who have successfully overseen previous promotional and preventive health projects.

Weaknesses

- Though involvement of high-level Ministry of Health investigators is great and demonstrates good buy in, the local investigators on the application can also be highly occupied to see day-to-day implementation of the project on the ground. Consideration for selected ART clinic manager(s) as co-investigators would be great.

3. Innovation:
Strengths

- The proposed work involves establishing smoking cessation interventions in ART settings, a unique strength that represents a shift in current clinical practice.
- The Insight™ platform is a technologic advance that is potentially scalable for administering cancer prevention behavioral interventions other than smoking cessation. A great technology as well that it has built in algorithms and branching logic that provides for personalized interventions based on individual characteristics including health conditions.

Weaknesses

- The Insight™ platform requires a smartphone and thus potentially excludes those without such phone.

4. Approach:
Strengths

- The research strategy is rigorous and uses appropriate methodology and well thought through analyses.
- There is adequate justification for the methods and analytical plan used.
- The team has adequately addressed data management plan from enrolment to post-trial dissemination.
- The timelines for project implementation are reasonable.

Weaknesses

- There is potential selection bias for participants enrolled in the intervention group of AA-MAP which requires a smartphone, and this has not been adequately addressed.

5. Environment:
Strengths

- The investigators from the University of Oklahoma Health Sciences Center and Moffitt Cancer Center have a demonstrable rich research environment with multiple platforms for multidisciplinary expertise and administrative support.
- The partnership of the US investigators with the Lao People's Democratic Republic Ministry of Health including the National Center for HIV/AIDS and STIs (CHAS) provides a reassurance for successful implementation of the proposed project and potential for scale up if results will be promising since policy makers are involved throughout the project design and implementation.
- The proposed project is designed to be implemented in established ART clinics and thus provides a unique strength of maximizing the HIV care delivery environment, a key element of the FOA.
- The use of the DHIS2 (dhis2.org) as the main electronic platform to manage ART patient demographic and medical information in Lao provides a unique opportunity for easy tracking of data of targeted study population.

Weaknesses

- Whereas the research environment for the investigators is excellent, it is unclear where exactly the Board of Regents of the University of Oklahoma Health Sciences Center fits in the Department of Family and Preventive Medicine especially that the PI (Bui) and other co-investigators are affiliates of this organization. The description of the Board of Regents is not adequately addressed except that it is in the Department of Family and Preventive Medicine.
- There is unclear evidence of the chosen ART clinics to conduct a randomized trial and ensure adherence to the protocol. The research infrastructure in Lao is not adequately described.
- The regulatory research environment for Lao People's Democratic Republic is unclear.

Study Timeline:
Strengths

- The project timeline is well described and includes key steps such as stakeholders' engagement meetings, fine-tuning study tools such as mHealth automated treatment program for use with Lao people with HIV.
- The project utilizes the existing DHISII, an electronic database which is already in use in the ART clinics, thus utilizing existing infrastructure.
- The project will be implemented through the existing human resources in ART clinic thus maximizing existing resources.

Weaknesses

- Regulatory submission and approval from all applicable IRBs i.e. Institutional Review Boards (IRBs) of the Lao National Ethics Committee for Health Research (NECHR) IRB #1 (IRB00006227) and of The University of Oklahoma Health Sciences Center (OUHSC) timeline has not been adequately included. Regulatory approvals can cause delays if not considered carefully in project planning.

Protections for Human Subjects: Acceptable Risks and/or Adequate Protections.

Data and Safety Monitoring Plan (Applicable for Clinical Trials Only): Acceptable.

Inclusion Plans:

- Sex/Gender: Distribution justified scientifically.
- Race/Ethnicity: Distribution justified scientifically.
- For NIH-Defined Phase III trials, Plans for valid design and analysis: Not applicable.
- Inclusion/Exclusion Based on Age: Distribution justified scientifically.

Vertebrate Animals: Not Applicable (No Vertebrate Animals).

Biohazards: Not Applicable (No Biohazards).

Applications from Foreign Organizations: Justified.

Select Agents: Not Applicable (No Select Agents).

Resource Sharing: Acceptable.

Authentication of Key Biological and/or Chemical Resources: Not Applicable (No Relevant Resources).

Budget and Period of Support: Recommend as Requested.

CRITIQUE 2

Significance:	2
Investigator(s):	1
Innovation:	1
Approach:	2
Environment:	1

Overall Impact: This is a well-written application that seeks to evaluate a practical approach for promoting smoking cessation among PWH in Laos. The team has laid out the public health benefits given high rates of smoking particularly in male PWH in Laos and has useful prelim data on desire to stop and intervention efficacy. Strengths of the application include the interventions selected – all EBIs, all pragmatic, likely feasible to integrate in busy HIV care clinics. The team is well qualified to lead this application given extensive directly relevant experience conducting related trials and obtaining preliminary data. The MPIs have complementary expertise and include an MPI from Laos and the intervention has involved careful collaboration in-country in development to date. The team incorporates post-doc training for Lao trainees. The research includes carefully specified outcomes that are the most relevant – reach/effectiveness and will involve a hybrid 2 cRCT in 8 clinics (with some prelim matching). Outcomes, sample size and approach are well described and the data informing sample size calculations is reasonable. Analysis approach is clearly laid out and biologic effectiveness will be assessed. Evidence that recruitment, timeline, and retention will be good. There is a good environment with appropriate data infrastructure and the team will leverage existing HIV care systems/DHIS2. PRISM IS outcomes will be evaluated using mixed methods and importantly detailed costing from governmental perspective is included. Weaknesses are minor – it is unclear if the program will be sustained in HIV care - there appears to be interest in a general population program with these elements. Reach estimates are insufficiently clear.

1. Significance:
Strengths

- The project could have a sustained impact on public health outcomes in Laos, specifically health outcomes among PWH who smoke.
- The team is proposing a novel combined strategy that is considered potentially feasible for the LMIC context with solid evidence of efficacy for individual components.
- The approach leverages HIV care system and could be integrated quite seamlessly – both mHealth and self-guided approaches exert minimal load on the system yet are of value to the participants.
- The study design includes components directly relevant to program leaders and policy makers including impact and IS outcomes, and importantly cost.
- The program could exert impact beyond HIV clinics – this is appealing to stake holders.

- A well-qualified team who has accrued relevant preliminary data and outlined a clear approach that is likely to be successful.
- The approach includes biologic outcomes of effectiveness.

Weaknesses

- Some lack of clarity regarding likelihood of long-term sustainment of the program.

2. Investigator(s):

Strengths

- MPIs bring complementary strengths from OUHSC, Laos and Moffit – all have prior experience conducting research on tobacco cessation and some have focus on HIV.
- Well detailed MPI plan outlining different roles and approaches to conflict/budget/communication.
- MPIs have previously worked together.
- One MPI is from Laos – this leadership structure enhances in-country ownership; in addition, the study has been co-conceived and has incorporated input from diverse in-country stakeholders.
- The team has also thought ahead to build in post-docs from Laos to increase capacity for research and expertise.
- Team is experienced in mhealth, clinical trials, analyses, and implementation science and has excellent experience in evaluating tobacco cessation approaches, including the ones in the proposed intervention.

Weaknesses

- None noted.

3. Innovation:

Strengths

- mHealth app for smoking cessation individually tailored and with evidence of effectiveness – built for Laos already in place – incorporating this app within HIV care is innovative.
- The combination approach of AAC (clinical interactions) and either MAP or SH are innovative and feasible strategies for Laos HIV care programs.
- Comprehensive IS approaches – hybrid 2 evaluation of effectiveness and reach and detailed PRISM outcomes will be useful.

Weaknesses

- None noted.

4. Approach:

Strengths

- The team has experience in similar tobacco cessation research in Laos with different populations (cancer/general) and can now link to the HIV care system.
- mHealth app and self-help option as well as the ACC clinical questions have all been demonstrated to have evidence of effectiveness, thus are EBIs that merit further examination for scale up.
- Laos tobacco control efforts at policy level are complementary and there is need for better programmatic cessation efforts within HIV care; preliminary engagement efforts note interest in the interventions.
- OUHSC team has developed effective mHealth app translated and appropriate for Laos that is ready to use; similarly self-help materials are available.
- Comparison of these two approaches (AAC-MAP vs. AAC-SH) is useful programmatically – one may have better reach (SH) and the other better effectiveness (SH) – making it hard to know which will have greatest impact – thus the study design to formally test both outcomes (reach/effectiveness) is useful for programs and policy makers.
- The outlined approach, design, and assessment of IS outcomes (PRISM) is clearly laid out.

- Effectiveness assessment with biologic marker is a strength.
- Reasonable sample size estimates for effectiveness – reach appears to be estimated upstream from the 1200 included – a little unclear; clearly laid out analytic approach for primary outcomes; past work has high retention and 6-month endpoints feasible; reasonable data for outcome estimates.
- Clearly laid out sites and volume – 8 clinics; good randomization strategy – balance by characteristics then randomized. Nicely outlined rationale for cRCT vs. stepped wedge or individual RCT.
- Nicely built app that includes personalization; AAC is feasible for implementation in clinical care and training plan outlined.
- Table 3 assessments clear and well outlined: qualitative work with health care workers also clear.
- Capacity building with training of Lao post-doc mentees is a great approach.

Weaknesses
- Long-term likelihood of this program being taken up is insufficiently clear – sustainment and barriers to implementation outside of research would be good to examine.
- Reach appears to be measured among those willing to stop smoking prior to enrollment – insufficiently clear with estimated 90% reach in SH and 70% for MAP based on smart phone access.
- Population of PWH is relatively small and long-term use of these methods in general clinic will be useful outside of HIV care as noted by stakeholders; however, the higher risk among PWH for smoking is clearly described so perhaps implementing in PWH then translating to general clinics is a good strategy.

5. Environment:
Strengths
- 3 MPIs at Oklahoma, Florida and in Laos – the three have collaborated on prior studies over the past several years; Having MPI from Laos strengthens LMIC leadership of application and team notes that the ideas have been collaboratively built from the start.
- All 3 environments have appropriate research infrastructure – unique mHealth and tobacco cessation programs and resources at OUHSC – mHealth Shared Resource which supports>40 studies, TSET Health Promotion Research Center.
- Moffitt is an NCI-designated comprehensive cancer center – includes biostat/data management, PRISM cores.
- Laos National Center for Laboratory and Epidemiology (NCLE) and MOH, National Tobacco Control Committee and National HIV/STI groups are also involved and have worked with the OUHSC team in the past; HIV care clinics use DHIS2 for ART and patient information – efficient data leverage options.

Weaknesses
- May be useful to increase community engagement.

Study Timeline:
Strengths
- Well detailed and likely feasible timeline – prep in Y1 and analysis in Y5 with cRCT Y2-4.
- Will leverage HIV care program clinical visits for enrollment, screening, and interventions.

Weaknesses
- None noted.

Protections for Human Subjects: Acceptable Risks and/or Adequate Protections.
- Well detailed HS considerations including risks, benefits, consent, confidentiality.

Data and Safety Monitoring Plan (Applicable for Clinical Trials Only): Acceptable.

Inclusion Plans:
- Sex/Gender: Distribution justified scientifically.
- Race/Ethnicity: Distribution justified scientifically.
- For NIH-Defined Phase III trials, Plans for valid design and analysis: Not applicable.
- Inclusion/Exclusion Based on Age: Distribution justified scientifically.
- Will have mostly male participants given the distribution of smoking among PWH in Laos. Will involve >18-year-olds.

Vertebrate Animals: Not Applicable (No Vertebrate Animals).

Biohazards: Not Applicable (No Biohazards).

Applications from Foreign Organizations: Justified.
- RFA addresses LMIC/HIV - and Laos has many PWH who could benefit from smoking cessation.

Select Agents: Not Applicable (No Select Agents).

Resource Sharing: Acceptable.

Authentication of Key Biological and/or Chemical Resources: Not Applicable (No Relevant Resources).

Budget and Period of Support: Recommend as Requested.

CRITIQUE 3

Significance:	2
Investigator(s):	1
Innovation:	2
Approach:	2
Environment:	1

Overall Impact: Smoking prevalence is high amongst males in Laos, and the co-investigator team has appropriate done the preliminary work to understand reasons for continuation. Currently, persons living with HIV who smoke in Laos currently have no access to smoking cessation support. The investigator team has developed an app that develop context-appropriate, interactive, tailored, personalized content (text messages, photos, and videos) regarding smoking cessation interventions and resources via a smartphone. Although the populations of individuals living with HIV in Laos is relatively small, the impact of this application is the cost-effectiveness analysis, which will be used to advocate for the intervention to be implemented nationwide, including in remaining ART clinics, HIV voluntary counselling and testing stations, and public hospitals.

1. Significance:
Strengths
- Of approximately 7.4 million citizens in Laos, 44%–51% of men and 7% of women smoke tobacco. There is currently no national tobacco treatment program or smoking cessation support in hospitals/clinics are available. This proposal is of a high public health importance.

- While the target vulnerable population of persons living with HIV is a modest size in Laos (17K), the investigator team has a much broader plan for dissemination of their smoking cessation intervention. As a result of their cost effectiveness analysis, they plan to advocate for the intervention to be implemented nationwide, including in remaining ART clinics, HIV voluntary counselling and testing (VCT) stations, and public hospitals. They have established appropriate collaborations with the Ministry of Health to advance this long-term goal.

Weaknesses
- None noted.

2. Investigator(s):
Strengths
- This application utilizes a 3-way MPI structure, triangulating between Laos, Moffitt Cancer Center, and OUHSC. Drs. Bui, Xangsayarath and Phandouangsy have worked together since 2017 in collaboration to analyze and publish national data regarding tobacco use in Laos.
- This application also includes investigators from 3 Lao governmental agencies within the MOH.
- Dr. Bui has a 20+ year history of collaboration in Vietnam, Laos, and Cambodia and currently is an MPI in a U01 study that uses similar mHealth technology to help Cambodian PWH quit smoking.
- The co-investigator team has a broad range of methodologic expertise.

Weaknesses
- None noted.

3. Innovation:
Strengths
- The Insight platform manages and delivers automatic, interactive, tailored smoking cessation content. It does not require cellular connectivity. Messages can be tailored personalized; however, it can function autonomously, minimizing human involvement. This intervention leverages that high phone ownership rates in Laos. It is potentially very affordable for large-scale implementation in LMICs.
- The Insight platform could potentially be used for messages regarding other cancer prevention behaviors.
- The investigators previously used the cultural adaptation of evidence-based interventions approach to translate, adapt, and validate our intervention content to the sociocultural context, language, and communication styles of Laotians during R21 phase.
- Preliminary data with focus groups identified factors affecting choices regarding smoking and quitting, which informed message development.

Weaknesses
- Pharmacologic approaches to smoking cessation are known to improve smoking cessation rates; however, they are currently inadequately available in the local context in Laos. This was adequately addressed (minor).

4. Approach:
Strengths
- The mHealth app has already been developed for the local context in Laos.
- The investigators provide reasonable justification for selection of the parallel cluster RCT design but will also allow all clinics the option to implement both interventions after the trial period.
- Aim 3 will employ the Cost-Assessment Methodology approach to conduct an assessment of the resources needed to widely implement the interventions and to quantify the potential gain from investing those resources from the perspective of the governmental service-providers. This specifically will address issues around generalizability scalability.
- The investigators incorporate several capacity-building elements into their approach, including in-service trainings for Insight and REDCap, online trainings in research, and opportunities for

colleagues from Laos to present the work at international conference and participate in manuscript and grant applications.

Weaknesses

- None noted.

5. Environment:

Strengths

- Of the 11 ART clinics in Laos, investigators plan to implement this project at 8 clinics in 5 provinces/regions that have highest overall numbers and prevalence of HIV+ populations.

Weaknesses

- There are only 17,000 people living with HIV in Laos, making this a relatively small target population (minor).

Study Timeline:

Strengths

- The study timeline is appropriately detailed, with adequate allocation of time for start-up and dissemination.

Weaknesses

- None noted.

Protections for Human Subjects: Acceptable Risks and/or Adequate Protections.

Data and Safety Monitoring Plan (Applicable for Clinical Trials Only): Acceptable.

Inclusion Plans:

- Sex/Gender: Distribution justified scientifically.
- Race/Ethnicity: Distribution justified scientifically.
- For NIH-Defined Phase III trials, Plans for valid design and analysis: Not applicable.
- Inclusion/Exclusion Based on Age: Distribution justified scientifically.
- The study will disproportionately enroll men. Development of the app did include strategies to extend the reach to and enrollment of women and sexual minorities.

Vertebrate Animals: Not Applicable (No Vertebrate Animals).

Biohazards: Not Applicable (No Biohazards).

Applications from Foreign Organizations: Justified.

- This proposal is in response to funding announcement RFA-CA-23-033 "Implementation Science for Cancer Control in People Living with HIV in Low- and Middle-Income Countries"; therefore, the foreign component is necessary and justified.

Select Agents: Not Applicable (No Select Agents).

Resource Sharing Plans: Acceptable.

Authentication of Key Biological and/or Chemical Resources: Not Applicable (No Relevant Resources).

Budget and Period of Support: Recommend as Requested.

THE FOLLOWING SECTIONS WERE PREPARED BY THE SCIENTIFIC REVIEW OFFICER TO SUMMARIZE THE OUTCOME OF DISCUSSIONS OF THE REVIEW COMMITTEE, OR REVIEWERS' WRITTEN CRITIQUES, ON THE FOLLOWING ISSUES:

PROTECTION OF HUMAN SUBJECTS: ACCEPTABLE. HS30. Potential risks are acknowledged, and adequate measures for protection are in place.

INCLUSION OF WOMEN PLAN: ACCEPTABLE. G1A. Both genders are included.

INCLUSION OF MINORITIES PLAN: ACCEPTABLE. M5A. Only foreign subjects.

INCLUSION ACROSS THE LIFESPAN: ACCEPTABLE. C3A. No children included.

COMMITTEE BUDGET RECOMMENDATIONS: The budget was recommended as requested.

Footnotes for 1U01CA294811-01; PI Name: Bui, Thanh C.

NIH has modified its policy regarding the receipt of resubmissions (amended applications).See Guide Notice NOT-OD-18-197 at https://grants.nih.gov/grants/guide/notice-files/NOT-OD-18-197.html. The impact/priority score is calculated after discussion of an application by averaging the overall scores (1-9) given by all voting reviewers on the committee and multiplying by 10. The criterion scores are submitted prior to the meeting by the individual reviewers assigned to an application, and are not discussed specifically at the review meeting or calculated into the overall impact score. Some applications also receive a percentile ranking. For details on the review process, see http://grants.nih.gov/grants/peer_review_process.htm#scoring.

MEETING ROSTER

National Cancer Institute Special Emphasis Panel
NATIONAL CANCER INSTITUTE
NCI Research Project-Cooperative Agreements (U01)

ZCA1 RPRB-M (M2)
03/13/2024

Notice of NIH Policy to All Applicants: Meeting rosters are provided for information purposes only. Applicant investigators and institutional officials must not communicate directly with study section members about an application before or after the review. Failure to observe this policy will create a serious breach of integrity in the peer review process, and may lead to actions outlined in NOT-OD-22-044 at https://grants.nih.gov/grants/guide/notice-files/NOT-OD-22-044.html, including removal of the application from immediate review.

CHAIRPERSON(S)

CREMER, MIRIAM, MD
PROFESSOR
DEPARTMENT OF OBSTETRICS AND GYNECOLOGY
CASE WESTERN RESERVE UNIVESITY
CLEVELAND, OH 44195

MORSE, GENE D, PHMD
DISTINGUISHED PROFESSOR AND DIRECTOR
DEPARTMENT OF PHARMACY PRACTICE
CENTER FOR INTEGRATED GLOBAL BIOMEDICAL SCIENCES
SUNY GLOBAL HEALTH INSTITUTE
UNIVERSITY AT BUFFALO
BUFFALO, NY 14203

MEMBERS

ANDERSON, JEAN, MD
PROFESSOR
DEPARTMENT OF GYNECOLOGY AND OBSTETRICS
JOHNS HOPKINS UNIVERSITY SCHOOL OF MEDICINE
BALTIMORE, MD 21287

CAMERON, JENNIFER ERIN, PHD
ASSOCIATE PROFESSOR
DEPARTMENT OF MICROBIOLOGY, IMMUNOLOGY
AND PARASITOLOGY
STANLEY S. SCOTT CANCER CENTER
LOUISIANA STATE UNIVERSITY HEALTH SCIENCES CENTER
NEW ORLEANS, LA 70112

CHINULA, LAMECK, MBBS, MD
ASSOCIATE PROFESSOR
DEPARTMENT OF OBSTETRICS AND GYNECOLOGY
GLOBAL WOMEN’S HEALTH
UNIVERSITY OF NORTH CAROLINA AT CHAPEL HILL
CHAPEL HILL, NC 27599

HUCHKO, MEGAN J., MD
ASSOCIATE PROFESSOR
DEPARTMENT OF OBSTETRICS AND GYNECOLOGY
DIRECTOR, CENTER FOR GLOBAL REPRODUCTIVE HEALTH
DUKE UNIVERSITY
DURHAM, NC 27708

JOHN-STEWART, GRACE, PHD, MD
PROFESSOR
DEPARTMENTS OF GLOBAL HEALTH, EPIDEMIOLOGY,
MEDICINE, AND PEDIATRICS
UNIVERSITY OF WASHINGTON
SEATTLE, WA 98104

MAIGA, MAMOUDOU, MD, PHD
ASSOCIATE PROFESSOR
DEPARTMENT OF BIOMEDICAL ENGINEERING
NORTHWESTERN UNIVERSITY
EVANSTON, IL 60208

REINHART, CRYSTAL, PHD
SENIOR RESEARCH SCIENTIST
CENTER FOR PREVENTION RESEARCH AND DEVELOPMENT
UNIVERSITY OF ILLINOIS
CHAMPAIGN, IL 61820

VAN LOON, KATHERINE, MD
PROFESSOR
DEPARTMENT OF MEDICINE
DIVISION OF HEMATOLOGY AND ONCOLOGY
UNIVERSITY OF CALIFORNIA, SAN FRANCISCO
SAN FRANCISCO, CA 94143

WARREN, EDUS HOUSTON, MD, PHD
PROFESSOR
DIVISIONS OF CLINICAL RESEARCH AND
VACCINE AND INFECTIOUS DISEASE
PROGRAM HEAD, GLOBAL ONCOLOGY
FRED HUTCHINSON CANCER RESEARCH CENTER
SEATTLE, WA 98109

WILKIN, TIMOTHY J., MD
PROFESSOR OF MEDICINE
DEPARTMENT OF MEDICINE
WEILL CORNELL MEDICAL COLLEGE
CORNELL UNIVERSITY
NEW YORK CITY, NY 10065

ZAHRBOCK, CARY, MSW
PRINCIPAL, CARY ZAHRBOCK CONSULTING
QUALITY IMPROVEMENT AND CANCER SURVIVORSHIP
NATIONAL COALITION FOR CANCER SURVIVORSHIP
CARY ZAHRBOCK CONSULTING
MINNEAPOLIS, MN 55418

SCIENTIFIC REVIEW OFFICER

GHALEB, AMR M., PHD
SCIENTIFIC REVIEW OFFICER
RESEARCH PROGRAM REVIEW BRANCH
DIVISION OF EXTRAMURAL ACTIVITIES
NATIONAL CANCER INSTITUTE, NIH
ROCKVILLE, MD 20850

EXTRAMURAL SUPPORT ASSISTANT

MILLER, DARNETT
PROGRAM SPECIALIST
RESEARCH PROGRAMS REVIEW BRANCH
DIVISION OF EXTRAMURAL ACTIVITIES
NATIONAL CANCER INSTITUTE
NATIONAL INSTITUTES OF HEALTH
ROCKVILLE, MD 20850

Consultants are required to absent themselves from the room during the review of any application if their presence would constitute or appear to constitute a conflict of interest.
